# Stabilization of Dicentric Translocations through Secondary Rearrangements Mediated by Multiple Mechanisms in *S. cerevisiae*

**DOI:** 10.1371/journal.pone.0006389

**Published:** 2009-07-28

**Authors:** Vincent Pennaneach, Richard D. Kolodner

**Affiliations:** 1 Ludwig Institute for Cancer Research, Departments of Medicine and Cellular and Molecular Medicine, and Moores UCSD Cancer Center, University of California San Diego School of Medicine, La Jolla, California, United States of America; 2 INSERM, Equipe Avenir, iRCM, CEA, F-92265, Fontenay aux Roses, France; National Cancer Institute, United States of America

## Abstract

**Background:**

The gross chromosomal rearrangements (GCRs) observed in *S. cerevisiae* mutants with increased rates of accumulating GCRs include predicted dicentric GCRs such as translocations, chromosome fusions and isoduplications. These GCRs resemble the genome rearrangements found as mutations underlying inherited diseases as well as in the karyotypes of many cancers exhibiting ongoing genome instability

**Methodology/Principal Findings:**

The structures of predicted dicentric GCRs were analyzed using multiple strategies including array-comparative genomic hybridization, pulse field gel electrophoresis, PCR amplification of predicted breakpoints and sequencing. The dicentric GCRs were found to be unstable and to have undergone secondary rearrangements to produce stable monocentric GCRs. The types of secondary rearrangements observed included: non-homologous end joining (NHEJ)-dependent intramolecular deletion of centromeres; chromosome breakage followed by NHEJ-mediated circularization or broken-end fusion to another chromosome telomere; and homologous recombination (HR)-dependent non-reciprocal translocations apparently mediated by break-induced replication. A number of these GCRs appeared to have undergone multiple bridge-fusion-breakage cycles. We also observed examples of chromosomes with extensive ongoing end decay in *mec1 tlc1* mutants, suggesting that Mec1 protects chromosome ends from degradation and contributes to telomere maintenance by HR.

**Conclusions/Significance:**

HR between repeated sequences resulting in secondary rearrangements was the most prevalent pathway for resolution of dicentric GCRs regardless of the structure of the initial dicentric GCR, although at least three other resolution mechanisms were observed. The resolution of dicentric GCRs to stable rearranged chromosomes could in part account for the complex karyotypes seen in some cancers.

## Introduction

The complex karyotypes observed in cancer cells have been shown to result from ongoing genome instability, in part triggered by dicentric chromosomes initiating bridge-fusion-breakage (BFB) cycles [1]–[3]. Other possible outcomes leading to the stabilization of dicentric chromosomes are centromere deletion, centromere inactivation and chromosome loss [4]. The anaphase bridges observed in a number of malignant tumors in early stages of carcinogenesis [4] have been proposed to result from telomere-telomere fusions induced by dysfunctional telomeres that are recognized and processed as double strand breaks (DSBs) resulting in the formation of dicentric chromosomes [5]–[9]. Consistent with this idea, the tumors observed in p53 defective mice with telomerase defects are characterized by numerous chromosomal rearrangements that recapitulate the classes of aberrant chromosomes observed in many solid tumors [8], [10]. Similarly, both spontaneous and induced sister chromatid fusions have been shown to result in karyotypic alterations in mammalian cells in cell culture [3], [11], [12]. In addition, a high frequency of chromosome end-to-end fusion has been described in cells from AT patients which have a defect in the checkpoint protein ATM [13] or from patients with Thiberge-Weissenbach syndrome [14].

Studies in *Saccharomyces cerevisiae* have been useful in identifying pathways that prevent the formation of gross chromosomal rearrangements (GCRs) and mechanisms by which GCRs are formed. Such studies have typically used assays that select for deletion of non-essential terminal regions of different chromosomes in haploid strains [15]–[17]. Using such assays, a broad spectrum of genes and pathways have been identified that play a role in suppressing GCRs [15], [17]–[26]. Structural analysis of GCRs and sequencing of GCR breakpoints have identified numerous types of monocentric GCRs including interstitial deletions, broken chromosomes healed by *de novo* telomere addition and non-reciprocal translocations as well as different dicentric GCRs including non-reciprocal translocations, chromosome fusions and isoduplication translocations [15], [16], [20], [27]–[30]. Monocentric and dicentric translocations and interstitial deletions appeared to consist of a broken chromosome that initially contained the genetic markers that were selected against joined to a fragment of the same or another chromosome, typically at regions of non-homology or very short homology. Consistent with such structures, the formation of these types of rearrangements was often dependent on non-homologous end-joining (NHEJ) [15], [27], [30]. Chromosome fusions appeared to result from the fusion of the counter-selected broken chromosome and an unprotected telomere of another chromosome [15], [16], [27], [31]. The formation of such chromosome fusions was highly dependent on NHEJ and is suppressed by telomerase and telomere protection pathways [27], [28], [30]. In contrast, dicentric isoduplications in which the broken chromosome was joined to a nearly identical copy of the broken chromosome in inverted orientation were predominantly found in homologous recombination (HR) proficient strains raising the possibility that HR was important for the resolution of dicentric GCRs to stable monocentric GCRs rather than being important for their initial formation [28], [30]. Small inverted repeats have also been shown to promote the formation of dicentric isoduplication translocations independently of Rad52 [32] and capped DSBs induced by processing of inverted repeats have been show to lead to GCRs through the initial formation of dicentric isoduplication translocations [33]. Yet other studies have identified GCRs mediated by apparent HR between Ty elements, although little is known about the pathways that form or prevent such GCRs [34]–[36].

Dicentric chromosomes have been shown to be unstable because the two centromeres are prone to being pulled into different daughter cells during mitosis [37], and studies in *S. cerevisiae* have provided insights into the fate of dicentric chromosomes. Engineered dicentric chromosomes have been shown to delete a centromere by HR between repeated sequences or by breakage and end-joining mediated deletion or alternatively such broken dicentric chromosomes can be healed by circularization, acquisition of a telomere or HR with another chromosome [38]–[41]. It has also been shown that the presence of inverted Ty elements or engineered inverted repeats can induce chromosome breakage resulting in a capped broken chromosome that can replicate to produce a dicentric chromosome much like a dicentric isoduplication [33], [35]. These dicentric chromosomes and dicentric chromosomes resulting from telomere-telomere fusion have been shown to break and be stabilized by either acquisition of a telomere [39], [40] or break induced replication (BIR) with another chromosome near Ty or delta elements resulting in a monocentric GCR [33], [36], [41]. Here we have analyzed the structure of spontaneous chromosome rearrangements isolated in different haploid telomerase deficient mutant backgrounds that were predicted to be dicentric GCRs based on the sequence of their primary translocation breakpoint [28], [30]. We found that all of the predicted dicentric GCRs were unstable and had undergone secondary rearrangements by a diversity of NHEJ- or HR-mediated events resulting in stable monocentric chromosomes; however, HR mediated events were found to predominantly contribute to the resolution of dicentric GCRs regardless of the primary rearrangement structure.

## Results

### Analysis of GCRs by pulse field gel electrophoresis (PFGE) reveals secondary rearrangement and circularization of predicted dicentric GCRs

In a series of previously published studies, we identified GCRs with a rearranged Can^r^ 5FOA^r^ chromosome V and in most cases characterized the rearrangement breakpoint at the DNA sequence level using a PCR mapping and DNA sequencing strategy. This allowed the identification of the nature and orientation of sequences present at the breakpoint relative to the parental chromosome sequences allowing prediction of the structure of the resulting rearranged chromosomes. In the current study we selected 21 GCRs for which the GCR breakpoints were previously analyzed [28] for further analysis including 2 predicted monocentric GCRs (M1-M2), 16 predicted dicentric GCRs (D1-D16) and 3 GCRs for which the breakpoint sequences could not be amplified (U1-U3). For 14 of these GCRs, the expected size of the rearranged chromosome V could be predicted from the breakpoint sequence (**Table 1**). The dicentric GCRs analyzed were selected from a collection of 141 previously published predicted dicentric GCRs that arose in a broad diversity of genetic backgrounds ([30], and unpublished data); a summary of the types of predicted dicentric GCRs and the genetic backgrounds in which they arose is presented in **Table S1**.

**Table 1:** Dicentric GCRs undergo resolution events independent of the primary GCR structure.

GCR strain:	primary rearrangement	predicted	observed	primary rearrangement^c^	class of secondary
genotype, isolate number^a^	breakpoint sequence^a^	GCR size	GCR size^b^	secondary rearrangement^c^	rearrangements^f^
				third rearrangement^c^	
M1: *rad51 tlc1* mut 23	non-reciprocal translocation	679 Kb	679 Kb	non-reciprocal translocation	–
M2: *rad55* mut 3	*de novo* telomere addition	539 Kb	539 Kb	*de novo* telomere addition	–
D1: *tel1 tlc1* mut 6	chromosome fusion	ND	540 Kb	*TELV* R fusion^d^	4
D2: *mec1 sml1 tlc1* mut 14	chromosome fusion	ND	540 Kb	*TELV* R fusion^d^	4
D3: *rad55 tlc1* mut 14	dicentric translocation	779 Kb	540 Kb	dicentric translocation,	3 then 4
				monocentric translocation,	
				*TELV* R fusion^d^	
D4: *mec1 sml1 tlc1* mut 3	dicentric isoduplication	1,082 Kb	740 Kb	dicentric isoduplication,	1
				monocentric translocation at *YELdelta4*	
D5: *mec1 sml1 tlc1* mut 15	dicentric isoduplication	1,085 Kb	580 Kb	dicentric isoduplication,	1
				monocentric translocation at *YELdelta1*	
D6: *mec1 sml1 lig4 tlc1* mut 21	dicentric isoduplication	1,070 Kb	1,300 Kb	dicentric isoduplication,	1
				monocentric translocation at *YELdelta4*	
D7: *mec1 sml1 lig4 tlc1* mut 22	dicentric isoduplication	1,085 Kb	745 Kb	dicentric isoduplication,	1
				monocentric translocation at *ura3-52*	
D8: *mec1 sml1 lig4 tlc1* mut 24	dicentric isoduplication	1,064 Kb	1,080 Kb	dicentric isoduplication,	1
				monocentric translocation at *ura3-52*	
D9: *mec1 sml1 lig4 tlc1* mut 26	dicentric isoduplication	1,065 Kb	860 Kb	dicentric isoduplication,	1
				monocentric translocation at *ura3-52*	
D10: *mec1 sml1 lig4 tlc1* mut 34	dicentric isoduplication	1,031 Kb	1,090 Kb	dicentric isoduplication,	1
				monocentric translocation at *ura3-52*	
D11: *rad59 tlc1* mut 7	chromosome fusion	1,485 Kb	800 Kb	chromosome fusion,	2
				dicentric translocation,	
				telomere capture^e^	
D12: *exo1 tlc1* mut 16	dicentric translocation	729 Kb	1,100 Kb	dicentric translocation,	2 then 2
				monocentric translocation,	
				monocentric translocation^e^,	
D13: *tel1 tlc1* mut 13	chromosome fusion	ND	1,500 Kb	chromosome fusion,	3
				CEN5 deletion	
D14: *rad59 tlc1* mut 2	dicentric isoduplication	1,072 Kb	540 Kb	dicentric isoduplication,	4
				chromosome fusion,	
				telomere capture^e^	
D15: *mec1 sml1 lig4 tlc1* mut 35	dicentric translocation	1,587 Kb	1,500 Kb	dicentric translocation,	ND
				centromere inactivation^e^	
D16: *tel1 tlc1* mut 12	chromosome fusion	ND	600 Kb	chromosome fusion,	ND
				telomere capture^e^	
U1: *mec1 sml1 tlc1* mut 6	ND	ND	1,400 Kb	dicentric isoduplication^e^	1
				monocentric translocation at *ura3-52*	
U2: *mec1 sml1 lig4 tlc1* mut 28	ND	ND	745 Kb	dicentric isoduplication^e^,	1
				multiple translocations at *YELdelta1* and *YELdelta4*	
U3: *xrs2 tlc1 mut 12*	ND	ND	840 Kb	dicentric isoduplication^e^,	1
				multiple translocations at *ura3-52* and *YELdelta4*	

a GCR containing strains were previously isolated and classified based on the sequence at primary GCR breakpoint as monocentric (M1 and M2), dicentric (D1-D16) or GCRs for which the breakpoint could not be amplified (U1-U3).

b Size of the chromosome V GCR was estimated using the chromosomes from the RDKY3615 strain as markers.

c Observed rearragments based on breakpoint rearrangement sequence, aCGH and PFGE data.

d The broken chromosome V is fused to chromosome V right telomere.

e Proposed rearragement based on aCGH analysis.

f The four classes of dicentric resolution events identified in this study are described in the “Discussion”.

ND, not determined.

**Figure 1:**
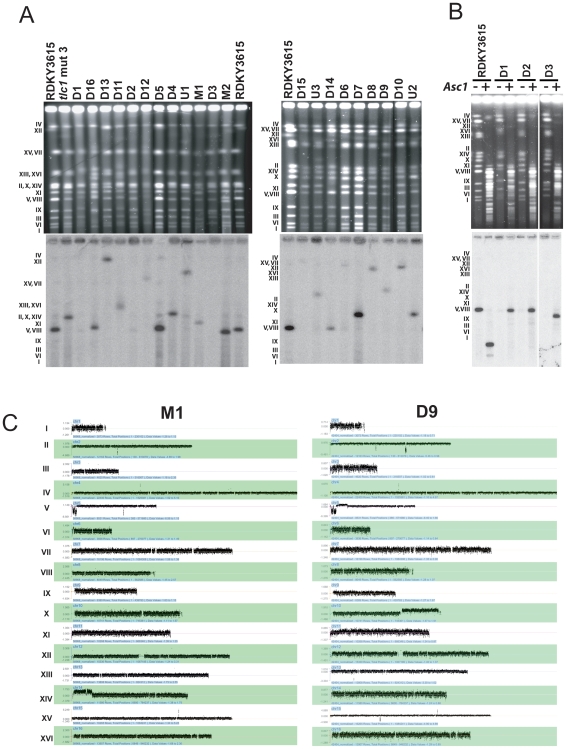
Karyotype analysis of 21 GCRs containing strains by PFGE and aCGH. (A) PFGE analysis of 21 Can^r^ 5FOA^r^ strains. Intact chromosomes from the indicated GCR strains were transferred to nitrocellulose membranes and hybridized to a radiolabeled chromosome V essential gene *YEL058W* probe. Rearranged chromosome V sizes were estimated relative to the sizes of chromosomes from the RDKY3615 wild-type and the CAN^r^ 5FOA^r^ *tlc1* mut 3 strain that were run as controls. (B) Analysis of circular chromosome V GCRs. Circular chromosome V GCRs were digested in the agarose plugs with *Asc* I for the indicated strains prior to PFGE. The intact and digested chromosome V was detected by hybridization with the *YEL058W* radiolabelled probe and the size of the resulting chromosome V fragment was estimated. (C) Karyotype analysis by aCGH of representative GCR containing strains is presented. The aGCH data of all GCRs analyzed in this study are present in the Figure S1. The normalized log_2_ ratio of the fluorescence intensities for each oligonucleotide relative to the reference strain is presented; in order to show all of the data points, it was necessary to use a different scale for the log_2_ ratio for each chromosome. Chromosome numbers are indicated to the left of the panel.

Chromosomes from each GCR containing strain were separated by PFGE and in most cases chromosomes with altered sizes were easily observed. These chromosomes were then analyzed by Southern blotting with a radio labeled chromosome V essential gene (*YEL058W*) probe to analyze the size of the rearranged chromosome V GCR (**Figure 1**) and the size of the rearranged chromosome V was estimated (**Table 1**). In only five cases (M1, M2, D8, D10 and D15) was the size of the rearranged chromosome V the same as the size of the rearranged chromosome predicted from the sequence of the rearrangement breakpoint. For 9 other GCRs, all predicted to be dicentric GCRs, the size of the rearranged chromosome V was different than that expected based on breakpoint sequence analysis. These observations suggested that most of the predicted dicentric GCRs were unstable and underwent secondary rearrangements.

PFGE analysis of three GCRs (D1, D2, and D3) did not detect a chromosome V related rearranged chromosome. As circular chromosomes are known to not enter pulsed-field gels [42], we performed in-plug digestion of the chromosomes with *Asc* I to cleave the single *Asc* I site present in chromosome V prior to PFGE analysis. As expected, *Asc* I digestion of the native linear chromosome V in the wild-type strain released a 163 Kb fragment detected by hybridization with the chromosome V specific probe (**Figure 1B**). Digestion of the D1, D2, and D3 GCRs resulted in the migration of a linear chromosome V into the gel consistent with these GCRs being circular chromosomes (**Table 1**). Breakpoint sequence analysis indicated that the D1 and D2 GCRs were chromosome fusions in which the broken left arm of chromosome V was fused to a telomere. These observations suggest that these two GCRs were formed by a break on the left arm of chromosome V that was healed by fusion to the telomere on the right arm of chromosome V resulting in a circular monocentric GCR; in each of these two cases the size of the linearized chromosome was consistent with this prediction (**Figure 1B**, and **Tables 1** and **2**). That the D3 GCR was maintained as a circular chromosome even though it was predicted to be a dicentric GCR with a chromosome V-XI fusion indicates that it underwent a secondary rearrangement resulting in circularization. The secondary rearrangements that resolved this dicentric GCR into a circular chromosome will be discussed below.

**Table 2:** Observed/predicted GCR structures.

GCR strain	observed GCR structure^a^	calculated GCR size	observed GCR size
M1	chr5[576869-32600];chr6[1-136720]	679 Kb	679 Kb
M2	chr5[576869-34989]; de novo telomere addition	539 Kb	539 Kb
D1	chr5[576869-39183]; circular chromosome	531 Kb	540 Kb
D2	chr5[576869-41273]; circular chromosome	529 Kb	540 Kb
D3	chr5[576869-33887]; chr11[430520-430679]; chr11[429861-429800]; telomere capture	541 Kb	540 Kb
D4	chr5[576869-34333]; chr5[36558-135612]; chr3[169569-316617]	789 Kb	740 Kb
D5	chr5[576869-34101]; chr5[34324-63728]; chr1[209439-230208]	593 Kb	580 Kb
D6	chr5[576869-41101]; chr5[42523-135612]; telomere capture	630 Kb	1,300 Kb
D7	chr5[576869-33010]; chr5[40036-116167]; chr14[102523-1]	722 Kb	745 Kb
D8	chr5[576869-41326]; chr5[47828-116167]; chr12[593147-1078175]	1,088 Kb	1,080 Kb
D9	chr5[576869-40594]; chr5[47982-116167]; ch10[472455-745741]	877 Kb	860 Kb
D10	chr5[576869-33220]; chr5[89383-116167]; ch12[599033-1078174]	1,055 Kb	1,090 Kb
D11	chr5[576869-32600]; chr16[944773-804641]; ch16[850625-944773]; telomere acapture	778 Kb	800 Kb
D12	chr5[576869-40594]; {chr14[589827-600226]; chr14[574092-525063]} repeated twice; chr14[567993-574092]; chr14[519164-1]	1,180 Kb	1,100 Kb
D13	chr5[576869-152307]; chr5[151667-33515]; chr15[1-1091289]	1,544 Kb	1,500 Kb
D14	chr5[576869-40709]; chr5[40361-40683]; telomere capture	536 Kb	540 Kb
D15	ND	ND	1,500 Kb
D16	ND	ND	600 Kb
U1	chr5[576869-36300]; chr5[36832-116167]; chr4[1095765-1531919]	1,492 Kb	1,400 Kb
U2	chr5[576869-41380]; chr5[41111-63728] repeated twice; chr5[63728-135612]; chr5[443393-576869]	780 Kb	745 Kb
U3	chr5[576869-42342]; chr5[42342-42788]; chr5[42788-116167] three repeats; chr5[116167-135612]; chr16[63006-1]	837 Kb	840 Kb

a Chromosome number is followed by the fragment SGD coordinates, fragments order and orientation reflect the proposed structure of the resolved GCR based on observed class of secondary rearrangements and observed chromosome fragments amplification by aGCH analysis.

b Size of listed chromosomes fragments were added to determine the calculated GCR size.

### Analysis of GCRs by array-Comparative Genomic Hybridization (aCGH)

The DNA from each of the 21 strains containing a GCR was also analyzed for copy number alterations by aCGH (**Figures 1**, **S1** and Array express accession numer E-TABM-732). Genomic DNA from each GCR containing strain was labeled with a Cy5 nucleotide and individually mixed with Cy3 labeled genomic DNA from the RDKY3615 wild-type strain followed by hybridization to an oligonucleotide array covering the entire genome. We confirmed the loss of the non-essential end of the chromosome V left arm containing both the *URA3* and *CAN1* genes in all 21 GCRs. The position of the GCR breakpoint on chromosome V identified by aCGH coincided exactly with the breakpoint positions previously determined by sequencing for all 18 GCRs.

The predicted monocentric M1 and M2 GCRs were examined as controls as these were expected to be stable GCRs. The M2 GCR was predicted to be a *de novo* telomere addition, and consistent with this aGCH analysis showed that the only karyotypic change present was a deletion of the left arm of chromosome V starting at the site of *de novo* telomere addition identified by sequencing (**Table 2** and **Figure S1**). The M1 GCR was predicted to be a monocentric translocation in which the broken chromosome V was joined to a telomere-containing fragment of the right arm of chromosome XIV. The aCGH analysis identified the deletion of the left arm of chromosome V starting at the observed breakpoint sequence and a duplication of the right arm of chromosome XIV from the chromosome XIV breakpoint sequence to the telomere (**Figure 1A** and **C**). The observed size of chromosome V GCR was consistent with that calculated for a monocentric translocation containing the broken chromosome V joined to the amplified segment of chromosome XIV (**Table 2**). The aCGH data and PFGE analysis were consistent with the M1 GCR strain also containing an intact copy of chromosome XIV (**Figure 1A** and **1C**) indicating that the M1 moncentric translocation was formed by a non-reciprocal mechanism.

The aCGH and PFGE data alone were insufficient to fully resolve the structure of the remaining GCRs. In each case, additional types of analysis were required to fully understand their structures. Below we describe the detailed analysis of these GCRs which has allowed us to resolve the structure of many of the GCRs, determine that all dicentric GCRs undergo some type of secondary rearrangement, and provide insights into the types of secondary rearrangements and the mechanisms by which they occur.

### Isoduplication GCRs undergo secondary rearrangements

**Figure 2:**
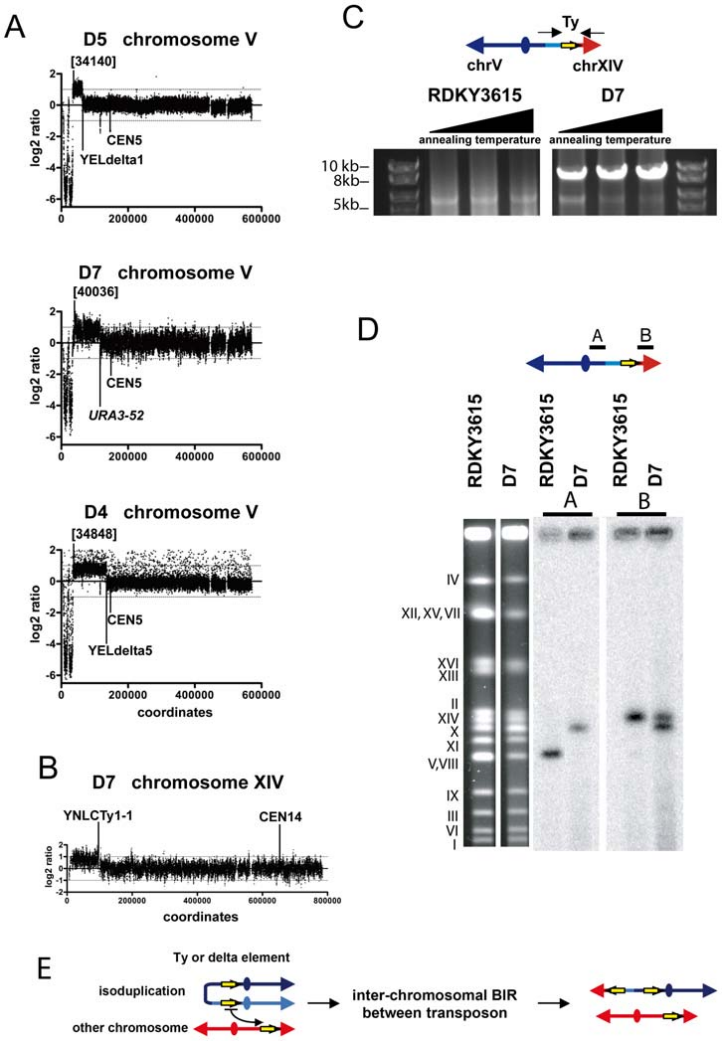
Analysis of representative examples of dicentric isoduplication GCRs. (A) aCGH analysis of chromosome V isoduplication GCRs indicates the presence of a deletion of a non-essential telomeric region of the left arm of chromosome V associated with duplication of an adjacent region of chromosome V. Numbers indicate the standard SGD nucleotide coordinates for the primary chromosome V duplication breakpoint. The second breakpoints are at *YELdelta1*, *ura3-52* and *YELdelta5* sequences in the examples presented. (B) aCGH analysis of chromosome XIV present in the D7 GCR strain is presented. The positions of the chromosome XIV centromere and the *YNLCTy1-1* element present at the chromosome XIV duplication breakpoint are indicated. (C) PCR analysis of the chromosome V:chromosome XIV non-reciprocal translocation Ty breakpoint region present in the D7 GCR strain. PCR reactions with a forward primer annealing to *YEL022W* on chromosome V and a forward primer annealing to *YNL286W* on chromosome XIV were performed with genomic DNA from RDKY3615 and the D7 strain. Three PCRs were performed with each DNA using annealing temperatures of 61.8°C, 63.8°C or 65°C, respectively. (D) Southern blot analysis of the chromosome V:chromosome XIV non-reciprocal translocation present in the D7 strain. The chromosomes from RDKY3615 and the D7 strain were separated by PFGE and stained with ethidium bromide. Hybridization with the radiolabelled *YEL058W* locus probe (probe A) revealed the native chromosome V in RDKY3615 and the rearranged chromosome V in the D7 strain. Hybridization with the radiolabelled *YNL286W* locus probe (probe B) revealed the native chromosome XIV in RDKY3615 and both native chromosome XIV and the duplicated region of chromosome XIV present at the end of the rearranged chromosome V present in the D7 strain; as expected, the rearranged chromosome detected by the chromosome V and chromosome XIV probes were the same size. (E) Proposed model for the resolution of the isoduplication by inter-chromosomal HR between repeat sequences, which can be Ty elements or delta sequences depending on the GCR analyzed.

We previously defined a class of GCRs, called isoduplication GCRs, in which a broken chromosome V is joined to a nearly identical fragment of chromosome V at the break site in the reverse orientation resulting in a dicentric chromosome V:chromosome V translocation [15], [28]. Seven such predicted dicentric isoduplication GCRs (D4–D10) were analyzed by aCGH. As expected, in all cases the non-essential region of the left arm of chromosome V was deleted and the deletion was associated with a duplication of a region of the left arm of chromosome V proximal to the site at which chromosome V was broken (three examples are shown in **Figure 2A**). The left arm sequences of chromosome V were only duplicated between the primary breakpoint junction with the second copy of broken chromosome V and a second breakpoint in the centromeric direction on the left arm of chromosome V (**Figures 1C** and **2**). The second breakpoint was at the Ty1 element causing the *ura3-52* mutation in the D7–D10 GCRs, at the *YELWdelta5* locus for the D4 and D6 GCRs and at the *YELWdelta1* locus in the D5 GCR. In addition, we observed that there was also duplication of an acentric region of another chromosome located between a Ty element and the telomere of the same chromosome arm in 6 of the 7 cases (D4–D5, D7–D10, **Table 2**; examples are shown in **Figures 1C**, **2B** and **S1**).

### Isoduplication GCRs undergo secondary non-reciprocal translocations mediated by repeated sequences

The D7 GCR was further analyzed to elucidate its complete structure. In this isoduplication GCR, the region of the left arm of chromosome V from the primary breakpoint to the Ty1 element at the *ura3-52* locus was joined to the broken left arm of chromosome V. In addition a 100 Kb region of chromosome XIV between the telomere and the Ty1 element *YNLCTy1-1* was duplicated (**Figure 2A** and **B**). Oligonucleotide primers that anneal to positions flanking *ura3-52* on chromosome V and on the duplicated chromosome XIV region bounded by the Ty1-1 element at the breakpoint were used in PCRs with genomic DNA from the D7 strain and the wild-type strain. A 10 Kb fragment was amplified from the genomic DNA of the D7 strain but not the wild-type strain. The size of the PCR fragment is consistent with the presence of a chromosome V:chromosome XIV breakpoint junction at the site of a Ty element (**Figure 2C**). Southern blot analysis with different probes specific for the chromosome V left arm and the chromosome XIV left arm sequences showed that duplicated chromosome XIV sequences comigrated with the rearranged chromosome V and demonstrated that the GCR containing strain also contained an intact copy of chromosome XIV (**Figure 2D**). These observations are consistent with a complex translocation mechanism (**Figure 2E**). The initial broken chromosome V was joined in inverted orientation to a second copy of itself resulting in a dicentric isoduplication GCR. The dicentric isoduplication GCR was then broken at or near the Ty1-1 element present at the *ura3-52* mutation 100 Kb away from the initial isoduplication breakpoint. The resulting chromosome V end was healed by copying of a region of the left arm of chromosome XIV from *YNLCTy1-1* to the telomere thereby generating a stable monocentric rearranged GCR. The size of the D7 GCR chromosome estimated by PFGE was consistent with the calculated size of this rearranged chromosome (**Table 2**). The presence of an intact copy of chromosome XIV in the GCR containing strain indicates that the second translocation was non-reciprocal consistent with the second rearrangement occurring by a Ty element mediated BIR event [43], although other mechanisms are possible [44], [45].

We did not examine the remaining 6 isoduplication GCRs (D4–D6, D8–D10) or the 3 suspected isoduplication GCRs (U1–U3) at this level of detail. However, all of these GCRs were associated with a second breakpoint located at either the Ty element causing the *ura3-52* mutation or a delta element (**Table 1**). With the exception of D6, all were also associated with duplication of a region of a second chromosome from a Ty element to a telomere (**Table 2** and **Figure S1**). The sum of the lengths of the broken chromosome V and the duplicated region of the second chromosome were consistent with the observed size of the GCR (**Tables 1** and **2**) and that an intact copy of the chromosome targeted by the second rearrangement was present. These findings taken together indicate that the secondary rearrangement mechanism resulting in the D7 GCR is a common mechanism (**Figure 2E**).

### Dicentric translocations and chromosome fusions also undergo secondary rearrangements at repeated sequences resulting in sequence amplification

**Figure 3:**
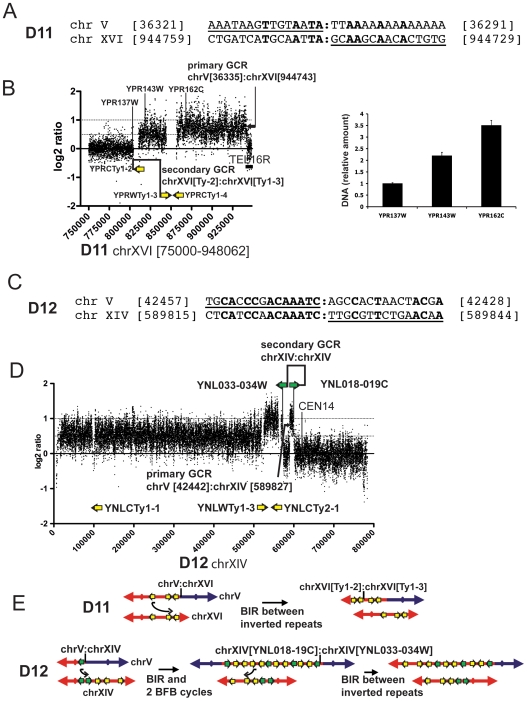
Analysis of GCRs with amplified sequences at the second breakpoint junction. (A) Primary breakpoint sequence of the D11 chromosome fusion. The underlined portion of the upper DNA sequence is the sequence of chromosome V at the breakpoint and the underlined portion of the lower sequence is the sequence of chromosome XVI at the breakpoint. Numbers indicate the standard SGD nucleotide coordinates for the indicated chromosome fragments. The nucleotides in bold are identical. (B) DNA copy number alteration along chromosome XVI of the D11 GCR determined by aCGH was validated by qPCR. The aCGH analysis of chromosome XVI from nucleotides position [750000] to [948062] is presented. The position of the chromosome V:chromosome XVI breakpoint is indicated. Arrows indicate the position and orientation of the Ty elements and *TEL16R* indicates the right telomere of chromosome XVI. Unique sequence primer pairs specific to chromosome XVI coding sequences *YPR137W*, *YPR143W* and *YPR162C* were selected to perform qPCR within the three chromosome XVI segments identified by aCGH. The relative amounts of genomic DNA determined by qPCR are expressed relative to the signal obtained from each primer pair with RDKY3615 control strain DNA. (C) Sequence at the breakpoint of the chromosome fusion of the D12 GCR. (D) DNA copy number alteration along chromosome XIV of the D12 GCR determined by aCGH analysis. Green arrows indicate the position of the tail to tail inverted repeats within the *YNL033-034W* and *YNL018-019C* region and the yellow arrows indicate *YNLWTy1-3* and *YNLCTy2-1* positions. *CEN14* refers to the chromosome XIV centromere position. (E) A model for the resolution of the D11 and D12 dicentric GCRs that proposes amplification by intra-chromosomal BIR between inverted repeats.

The aCGH analysis of two GCRs, the D11 predicted chromosome fusion GCR and the D12 predicted dicentric translocation GCR, revealed amplification of regions bounded by repeated sequences associated with the secondary rearrangement. The D11 GCR was predicted from its primary breakpoint sequence to be a chromosome fusion where the broken chromosome V was fused to the right telomere of chromosome XVI (**Figure 3A**). The aCGH analysis showed that two regions of chromosome XVI were amplified. A duplicated 34 Kb region was observed that was flanked by the *YPRCTy1-2* element on one side and the inverted *YPRWTy1-3* and *YPRCTy1-4* elements on the other side. In addition, a 92 Kb region of chromosome XVI bounded by the *YPRWTy1-3* and *YPRCTy1-4* elements and the right telomere was triplicated. Analysis of three loci along the right arm of chromosome XVI by qPCR confirmed these copy number changes (**Figure 3B**). In addition, PFGE analysis indicated that the GCR containing strain also contained an intact copy of chromosome XVI as well as the rearranged chromosome V. The rearranged chromosome V had a size (**Table 1**) consistent with the size of a chromosome containing the broken chromosome V, one copy of the duplicated chromosome XVI region and two copies of the triplicated chromosome XVI region (**Table 2**).

Breakpoint sequence analysis of the D12 GCR indicated that a broken chromosome V was fused to position [589921] of the centromere containing chromosome XIV fragment resulting in a predicted dicentric translocation (**Figure 3C**). The aCGH analysis indicated the presence of four copies of the region of chromosome XIV between nucleotide [589921] and the ∼4.2 Kb *tI(AAU)N2-YNL018C-*YNL019C region (*YNL018-019C* region), four copies of the region between the Ty elements *YNLWTy1-2* and *YNLCTy2-1*, three copies of the region between the *tI(AAU)N1-YNL034W-YNL033W* region (*YNL033-034W* region) and *YNLCTy2-1*, as well as the presence of two copies of the region of chromosome XIV between the left telomere and *YNLCTy1-2* (**Figure 3D**). Interestingly, the 4.2 Kb chromosome XIV *YNL018-019C* and *YNL033-034W* regions are nearly identical and are in an inverted orientation relative to each other. The size of the GCR chromosome determined by PFGE (**Table 1**) was consistent with a rearranged chromosome containing the broken chromosome V, three copies of the chromosome XIV region between positions [589921] and the *YNL018-019C region*, three copies of the region between the *YNL033-034W* region and *YNLCTy2-1* and one copy of the chromosome XIV region between *YNLWTy1-2* and the left telomere (**Table 2**). In addition, PFGE analysis indicated that the GCR containing strain also contained an intact copy of chromosome XIV.

Although we did not determine the exact structure of the D11 and D12 GCRs, the structural information obtained does make it possible to hypothesize how GCRs containing the amplified regions were formed. In the case of the D11 chromosome V:chromosome XVI right telomere fusion GCR, it is likely that this dicentric GCR broke at or near *YPRCTy1-2* followed by a BIR event mediated by the *YPRCTy1-3* in inverted orientation copying the 92 Kb region from the *YPRWTy1-3* element to the telomere of another copy of chromosome XVI resulting in a GCR containing the broken chromosome V, one copy of the 34 Kb region of chromosome XVI and two copies of the 92 Kb terminal region of chromosome XVI (**Figure 3E**). The pattern of amplifications observed in the case of the D12 GCR could be explained by a complex mechanism initiating by the original chromosome V:chromosome XIV dicentric translocation breaking within the *YNL018-019C* region and the resulting end initiating a BIR event by priming in the inverted repeated *YNL033-034W* region on the same arm of chromosome XIV. DNA synthesis would then copy toward the telomere followed by template switching within the inverted Ty duplication *YNLWTy1-2*, and *YNLCTy2-1* from the *YNLWTy1-2* to the *YNLCTy2-1* followed by a second template switch within the inverted *YNL033-034W* and *YNL018-019C* region present at the primary breakpoint junction from the *YNL033-034W* to the *YNL018-019C*, and then followed by copying to the end of chromosome V to yield a dicentric isoduplication. This dicentric chromosome would then undergo two BFB cycles in which the breakage events were induced by the inverted repeats [33] present in the dicentric chromosomes to amplify from the *YNLWTy1-2* element to *YNL018-019C* region; critically, this amplification mechanism would account for the partial internal deletion present between *YNL033-034W* and *YNL018-019C*. Finally, one additional breakage cycle would occur at the amplified region allowing a BIR event to be initiated by a DSB in a *YNLWTy1-2* element to prime synthesis at the intact chromosome XIV *YNLWTy1-2* element to the telomere accounting for the duplication of this last region (**Figure 3E**). Our data indicate that regions bounded by inverted sequences can be subject to amplification during the events by which the GCRs are formed. Multiple BFB cycle and/or BIR with possible template switching could contribute to the observed amplifications. In other studies, similar amplifications have been observed to be initiated by DSBs induced at engineered inverted repeats [35], [46], [47]. However, in the cases analyzed here, clearly the inverted repeat sequences could not have mediated the initial breakage events that initiated the formation of the primary dicentric GCRs.

### Resolution of a dicentric GCR by deletion of a centromere

**Figure 4:**
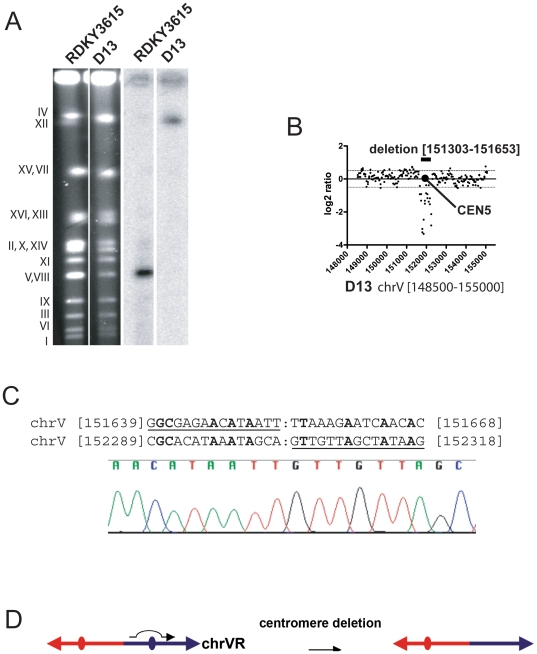
Resolution of the D13 dicentric GCR by deletion of a centromere. (A) Southern blot analysis of RDKY3615 and D13 GCR DNA using the radiolabelled chromosome V essential gene *YEL058W* probe. (B) The aCGH analysis of the chromosome V [148000-154000] region is presented. The deletion region and the chromosome V centromere are highlighted and the standard SGD coordinates of the deleted nucleotides are indicated. (C) Breakpoint sequence analysis of the chromosome V centromere deletion. Fragments amplified by PCR from RDKY3615 and D13 GCR genomic DNA with primers CEN5F (TTTTTGTGAATTAGGGAACGGAAGG) and CEN5R (TCGATGAATACAGACATTGAATAGC) and then sequenced. The sequence chromatogram of the PCR product from the D13 GCR DNA is presented. (D) Model for the resolution of the D13 dicentric GCR by interstitial deletion of a chromosome V region containing *CEN5*.

Prior breakpoint sequence analysis of the D13 GCR indicated it was a fusion between a broken chromosome V and the telomere of another chromosome, and consistent with this PFGE analysis revealed that the size of this rearranged chromosome was 1,500 Kb (**Figure 4A** and **Table 1**). Analysis by aCGH revealed a 351 bp deletion including the chromosome V centromere (**Figure 4B**), and PCR amplification and sequencing of this deletion region revealed a breakpoint at a region of non-homology (**Figure 4C**); no sequences from any other chromosome were amplified or deleted (**Figure S1**). These data are consistent with a model where the centromere-containing fragment of a broken chromosome V fused to the telomere of another chromosome resulting in a dicentric chromosome followed by deletion of the chromosome V centromere stabilizing the rearranged chromosome (**Figure 4D**). We note that if chromosome V was fused to chromosome XV, the size of this predicted GCR would be close to the size observed (**Table 2**) but we did not investigate this possibility further.

### Resolution of dicentric GCRs can be associated with multiple BFB cycles

**Figure 5:**
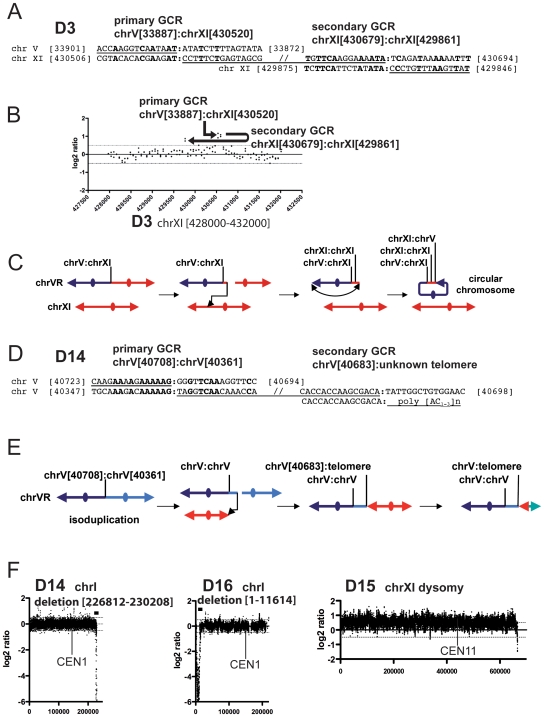
Complex karyotypes are associated with the resolution of dicentric GCRs. (A) Sequence observed at the first and second rearrangement breakpoints of the D3 GCR resulting in the formation of a circular GCR. (B) aGCR analysis of the chromosome XI region containing the secondary GCR breakpoints. The positions of the chromosome V:chromosome XI junction and the chromosome XI:chromosome XI secondary GCR breakpoints are indicated, with the arrows indicating the orientation of the junctions observed by breakpoint sequence analysis. (C) Model for the formation of the D3 GCR where multiple events have resulted in the resolution of the primary dicentric GCR. The position of the chromosome V:chromosome XI, first chromosome XI:chromosome XI and second chromosome XI:chromosome XI breakpoints are indicated. (D) Sequence observed at the first and second rearrangement breakpoints of the D14 GCR. (E) Model for the formation of the D14 GCR where multiple events have resulted in the resolution of the primary dicentric GCR. The positions of the first and second chromosome V:chromosome V breakpoints are indicated. (F) Examples of the aCGH analysis of selected chromosomes showing copy number changes observed in strains with chromosome V GCRs. In each example the nature of the karoytype modification is indicated followed by the standard SGD coordinates of the involved region. Chromosome I in both the D14 and D16 GCR containing strains had a terminal deletion in a region containing non-essential genes. The D15 chromosome V GCR is associated with whole chromosome XI dysomy.

Breakpoint sequencing of the D3 and D14 GCRs revealed the sequence signature of two successive predicted dicentric rearrangements, followed by the sequence signature of a subsequent rearrangement resulting in a monocentric GCR. Based on the sequence of the primary GCR breakpoint, the D3 GCR was predicted to be a dicentric translocation where the broken chromosome V was fused to a fragment of chromosome XI. However, the chromosome V breakpoint sequence analysis revealed that the first breakpoint was associated with a second downstream breakpoint whereby a 159 bp chromosome XI fragment [430520-430679] was fused to the same chromosome XI arm in the opposite orientation at a region of microhomology at position [429860] (**Figure 5A**). This region appeared to contain both duplicated and triplicated sequences by aCGH although we did not confirm this by qPCR. The sequence orientation at the second breakpoint predicts that this rearrangement would result in a monocentric GCR containing a duplication of the sequences of chromosome XI from position [429860] to the telomere. However, the aCGH analysis showed that only a 60 bp region of chromosome XI was amplified at this breakpoint (**Figure 5B**). The calculated size of such a GCR structure is consistent with the size determined for the circular D3 GCR (**Tables 1** and **2**). The observation that the D3 GCR is a circular chromosome (**Figure 1B**) therefore indicates that this GCR must have broken again between the last breakpoint and the telomere, followed by joining of the broken end to the right telomere of chromosome V (**Figure 5C**). The PFGE data indicated that an intact copy of chromosome XI was present in the strain containing the D3 GCR, and the aCGH data revealed no copy number changes for chromosome XI sequences other than the amplifications at the breakpoint junctions. The sequence of the primary breakpoint revealed that D14 GCR was a dicentric isoduplication (**Figure 5D**). The broken chromosome V was fused to an inverted 320 bp chromosome V fragment telomere-distal to the primary breakpoint, and the inverted chromosome V fragment was then fused to the telomere of an unidentified chromosome (**Figure 5E**). The observed size of this rearranged chromosome V is consistent with the site of telomere fusion being at or near the end of the GCR suggesting that a telomere was added at this site, possibly by HR after breakage of the resulting dicentric GCR near the site of the chromosome fusion (**Figure 5E** and **Table 2**). Unlike the cases of the D4–D10 dicentric isoduplication GCRs for which the second breakpoints were in repeated genomic sequences, the second breakpoints in the D3 and D14 GCRs were at non-homology or microhomology sequences. In addition, the fact that the sequence at the second breakpoint in D3 and D14 GCRs predicts the formation of a dicentric GCR suggests that multiple rounds of BFB could be associated with the healing of the original broken chromosome V.

### Resolution of dicentric GCRs can be associated with multiple karyotypic changes

Additional karyotypic changes identified by aCGH include single chromosome disomy of a chromosome that was unrelated to the GCR analyzed (an example is chromosome XI in the D15 GCR strain), terminal deletion of non-essential genes (examples are chromosome I in the D16 and D14 GCRs), and chromosome fragment amplification at a breakpoint sequence containing a genomic repeat (an example is chromosome XVI in the D15 GCR strain) or at a breakpoint at single copy sequences (an example is chromosome VII in the D12 GCR strain) (**Figures 5F** and **S1**). Because the additional karyotypic alterations were unique to each GCR, we believe that these events occurred independently during selection of the Can^r^ 5FOA^r^ GCR (compare the aCGH analysis of GCRs selected from the same Can^s^ 5FOA^s^ parental strain (see MATERIALS and METHODS). However, our results do not prove that these karyotypic changes did not result from the events that resolved the initial dicentric chromosome V GCR. Overall these observations illustrate that complex karyotypic changes can occur in a limited number of generations after the initial selected GCR was formed.

We additionally analyzed three GCRs (U1–U3) in which we were unable to predict the rearrangement structure, because we could locate, but not determine the primary chromosome V breakpoint sequence. In the case of the U1 GCR (**Figure S1**) duplication of a break proximal region of chromosome V, suggested that the initial event in the formation of the U1 GCR resulted in the formation of a dicentric isoduplication. In the case of the U2 and U3 GCRs, we also observed duplication, triplication, and quadruplication of regions of both arms of chromosome V associated with the deletion of the chromosome V left arm fragment including duplication of an initial break proximal region of chromosome V suggestive of an initial isoduplication intermediate (U3 is shown in **Figures 5F** and **S1**, and **Table 2**). The breakpoints of these amplified regions were at delta sequences and at the Ty1-1 element at the *ura3-52* locus suggesting that these GCRs might have resulted from BFB cycles that occurred after the formation of an initial chromosome V isoduplication GCR. All three of these GCRs were also associated with duplication of the end of another chromosome or the right end of chromosome V from a Ty element to a telomere. As a consequence, it is likely that the stable rearranged U1, U2 and U3 GCRs had the same types of structures and were formed by the same mechanisms as the other GCRs predicted to be dicentric by breakpoint sequence analysis (**Table 2** and **Figure S1**).

### HR is a major pathway for resolution of dicentric GCRs

**Figure 6:**
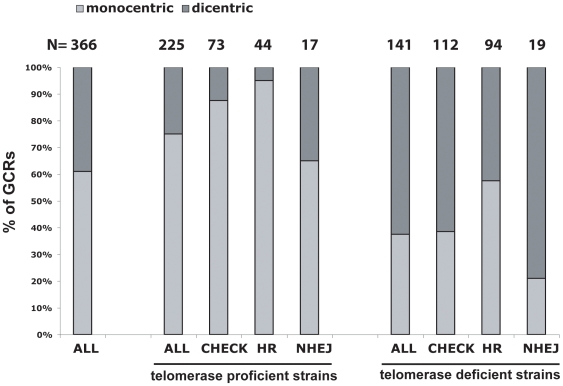
HR defects are associated with decreased frequencies of predicted dicentric GCRs. A total of 366 events were analyzed including 225 predicted monocentric GCRs (*de novo* telomere addition GCRs where excluded from this analysis) and 141 predicted dicentric GCRs; these GCRs are described in [30]. The percentage of predicted monocentric GCRs and dicentric GCRs were determined for each indicated group of strains. Telomerase deficient includes all strains that contain *tlc1* or *est2* mutations. CHEK includes strains that contain *chk1*, *dun1*, *mec1*, *mec3*, *pds1*, *rad9*, *rad53* and/or *tel1* mutations, REC includes strains that contain *rad51*, *rad52*, *rad54*, *rad55*, *rad59* and/or *rdh54* mutations and NHEJ includes strains that contain *lig4*, *ku70*, *ku80* or *mre11* mutations. Strains containing other mutations that might affect these different pathways were not included in this analysis. Numbers above the histogram indicate the actual number of GCRs in each group.

The aCGH analysis presented above revealed that in 11 of the 16 predicted dicentric GCRs, the primary rearrangement was associated with at least one additional rearrangement for which the second breakpoint was in a genomic region containing a repeated sequence. In a collection of 366 translocations identified by breakpoint sequence analysis ([30], and unpublished data), 141 GCRs were predicted to be dicentric with 40% being found in telomerase proficient strains and 60% being found in telomerase deficient strains (**Figure 6**). Defects in HR pathways were associated with a significant reduction of the frequency of predicted dicentric GCRs to 5% (p = 0.002; Fisher Exact test) and to 40% (p = 0.016; Fisher Exact test) in telomerase proficient and telomerase-deficient strains, respectively. Similarly, checkpoint defects were associated with a significant reduction in the frequency of dicentric GCRs to 12% (p = 0.022; Fisher Exact test) in telomerase proficient strains. The other apparent changes in the distribution of predicted dicentric GCRs were not significant. It was previously suggested that most dicentric GCRs (with the exception of some isoduplications) were formed by end joining mechanisms because the primary breakpoints were at regions of non-homology or microhomology [30]. A possible explanation for these observations is that NHEJ or some type of microhomology-mediated recombination [48]–[50] often plays a role in forming the initial monocentric and dicentric translocation breakpoint and HR-pathways more efficiently promotes the secondary rearrangements that stabilize the GCRs. In the absence of HR, dicentric translocations can form, however breakage of the dicentric GCRs in the absence of efficient secondary rearrangement mechanisms might lead to a high frequency of cell death explaining the decrease of predicted dicentric GCRs observed in HR-deficient strains.

### Extensive chromosome end decay in mec1 sml1 tlc1 mutants

**Figure 7:**
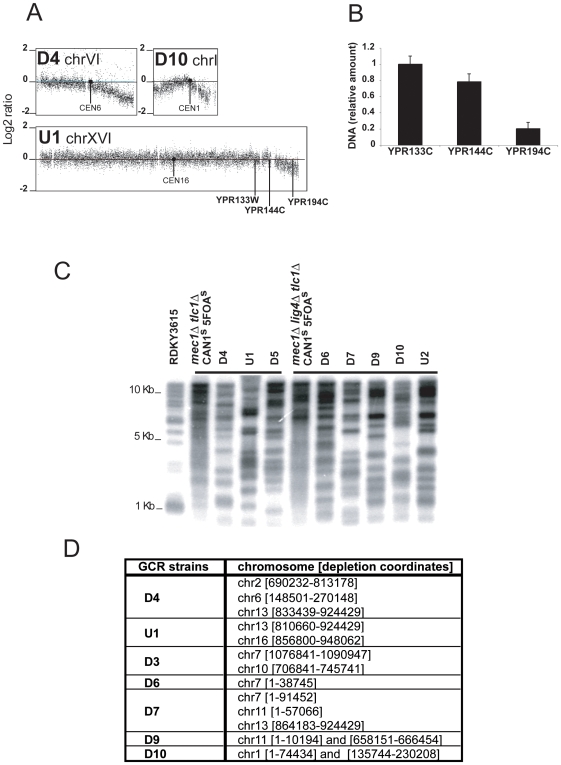
Degradation of chromosome ends in strains containing *mec1* and *tlc1* mutations. (A) aGCH analysis of representative chromosomes with signal decay at their ends is presented. (B) The qPCR validation of the decrease in the amount of genomic DNA on the right arm of chromosome XVI in the U1 GCR strain using primer pairs specific to *YPR133W*, *YPR144C* and *YPR194C loci*. The relative amounts of genomic DNA determined by qPCR at the *YPR133W*, *YPR144W* and *YPR194C* loci in the U1 strain are expressed relative to the signal obtained with each primer pair using RDKY3615 control DNA. (C) Telomere lengths in a RDKY3615 Can^s^ 5FOA^s^ strain, a *mec1 sml1 tlc1* Can1^r^ 5FOA^r^ post-senescence strain, a *mec1 sml1 lig4 tlc1* Can1^s^ 5FOA^s^ post-senescence strain, *mec1 sml1 tlc1* GCR strains (D4, U1 and D5) and *mec1 sml1 lig4 tlc1* GCR strains (D6, D7, D9 and D10) were analyzed by Southern blot with a poly(C_1-3_/TG_1–3_) radiolabelled probe hybridized to *Xho* I-digested genomic DNA. (D) Chromosomes with at least one degraded end are listed. The chromosome number is followed by the standard SGD nucleotide coordinates of the chromosome region that has a negative log_2_ ratio.

In addition to the presence of the primary GCR, we observed an unexpected class of aberrant chromosomes in all *mec1 sml1 tlc1* and *mec1 sml1 lig4 tlc1* strains analyzed. The aCGH analysis showed that in each of these mutants there appeared to be a loss of DNA at the ends of one to three chromosomes in the population of cells from which the DNA was isolated (three examples are shown in **Figure 7A**). The regions of chromosome loss were heterogeneous and extended for as much as 123 Kb from the telomere and included regions containing essential genes (**Figure 7A** and **D**). The loss of chromosome ends was also verified by qPCR; for example, analysis of loci on the right arm of chromosome XVI showed that on average only 20% of single copy genomic DNA was present 22 Kb from the telomere when the cell population was harvested (**Figure 7B**). Analysis of genomic DNA from the *mec1 sml1 tlc1* and *mec1 sml1 lig4 tlc1* GCR mutants by digestion with *Xho* I and hybridization with a telomeric probe indicated that in all cases the telomeres were consistent with post-senescence Type II survivors [51], although there appeared to be an increase in the number of faster migrating fragments in the D4, U1 and D5 GCRs compared to the *mec1 sml1 tlc1* CAN^s^ 5FOA^s^ Type II parental strain and in the D6, D7, D9 D10 and U2 GCRs compare to the *mec1 sml1 lig4 tlc1* CAN^s^ 5FOA^s^ Type II parental strain (**Figure 7C**). These observations suggest that some telomeres are shorter in *mec1 sml1 tlc1* mutant strains and that other telomeres are ultimately completely lost leading to degradation and shortening of the chromosomes as the cells divide during propagation of the culture.

## Discussion

Here we characterized the karyotypic alterations associated with 21 primary GCRs selected for loss of the left arm of chromosome V [28]–[30] through a combination of breakpoint sequence analysis, aCGH analysis and PFGE analysis. These studies established a number of findings. First we confirmed the primary structure of both monocentric and dicentric GCRs predicted from breakpoint sequence analysis. Second, all of the dicentric GCRs were unstable and were resolved to monocentric GCRs by at least four different mechanisms independent of the structure of the initial dicentric GCR. Third, both HR and NHEJ were found to mediate the resolution of dicentric GCRs, although HR events were the most frequently observed resolution mechanism. Finally, we observed chromosomes with telomere-free ends and found that a Mec1-dependent function contributes to the protection of chromosome ends from extensive degradation and likely facilitates the maintenance of telomeres by HR in the absence of telomerase.

### Multiple approaches are required to fully characterize karyotypic alterations associated with GCRs

aCGH provided a considerable amount of information that could not always be obtained using other methods, which was useful in studying the structure of GCRs. Consistent with the properties of the GCR assay that selects for loss of the left arm of chromosome V, aCGH confirmed that a telomeric fragment of the left arm of chromosome V was deleted in all 21 GCRs analyzed by aCGH. The aCGH data confirmed the breakpoint position on the left arm of chromosome V for all 18 GCRs in which the breakpoint was previously identified by sequencing and identified the chromosome V breakpoint position for the 3 GCRs for which the breakpoint had not been previously sequenced. We observed terminal deletion of a non-essential end of three other chromosomes besides chromosome V in three GCR containing strains, supporting the hypothesis that spontaneous terminal deletions can target the non-essential ends of any of the 16 chromosomes in haploid *S. cerevisiae* [15]–[17], [27]. aCGH also allowed detection of other more subtle features of genome instability including amplification of regions of DNA at GCR breakpoints, whole chromosome disomy and chromosomes being maintained without telomeres with associated degradation of the chromosome ends.

However, with the exception of the monocentric GCRs and the *de novo* telomere addition GCRs analyzed, aCGH alone could not resolve the structure of the predicted GCRs, and the complete analysis of these GCRs required additional information obtained through chromosome size determination by PFGE, PCR mediated breakpoint amplification, qPCR to determine copy number, breakpoint sequencing and Southern blot analysis, aCGH data played a critical role in designing the most efficient strategies to apply these different methods. Ultimately, using these methods, it was possible to deduce the structure of most of the dicentric GCRs analyzed, although we did not determine the structure of all of these GCRs to the nucleotide sequence level.

### Four classes of secondary rearrangements are associated with the resolution of dicentric GCRs

Previous studies using engineered dicentric chromosomes demonstrated that dicentric chromosomes are unstable [38]–[41], [52]–[54], most likely due to mechanical breakage [55], [56] during cell division as a result of assembling spindles to more than one centromere per chromosome. Consistent with these prior observations, our analysis revealed that the formation of an initial dicentric GCR leads to secondary rearrangements for 13 of the dicentric GCRs analyzed. Four classes of secondary rearrangements were identified providing expanded insights into the processes by which dicentric chromosomes are resolved and the diversity of complex GCRs that can result through the formation of an initial dicentric GCR.

In the first class of dicentric resolution events, the second breakpoint occurred at a Ty or a delta element located between the two centromeres and in most cases was associated with the partial duplication of a region of another chromosome from a Ty or delta sequence to the telomere of the same chromosome arm. In all cases, an intact copy of the chromosome targeted by the second breakpoint was present indicating that the second rearrangement was a non-reciprocal translocation. The structures of the chromosomes present were consistent with the second translocation being formed by BIR between transposon elements, although other mechanisms are also possible [43]–[45]. Such events promoted the resolution of most of the dicentric isoduplications studied. Our previous studies indicated that the formation of dicentric isoduplication GCRs was dependent on HR even though the primary breakpoint sequences were found at regions of non-homology or microhomology [30]. Our present results suggest that one reason for the dependence of these dicentric translocations on HR is that the most efficient events that result in loss of one of the two centromeres stabilizing the dicentric GCR depend on HR. However, the observation of isoduplication GCRs with secondary rearrangement breakpoints at regions lacking homology suggests that HR-independent secondary rearrangements can also occur, albeit at lower rates. Similarly, other studies have shown that HR between transposon elements can mediate the conversion of broken dicentric chromosomes to rearranged monocentric chromosomes [33], [35], [41].

In the second class of dicentric resolution events, the second breakpoint was located at the site of inverted repeats located between the centromeres and was associated with translocation to another chromosome at the site of related sequences and amplification of regions bounded by the repeat sequences. The second translocation events were also non-reciprocal. Amplification of sequences bounded by repeats in inverted orientations has been seen in other studies [33], [57]. We suggest that in the case of this class of secondary rearrangements the dicentric GCR broke at or near the repeated sequence. The observation of inverted repeat sequences at the site of the second breakpoint raises the possibility that these inverted repeat sequences induced the breakage of the initial dicentric GCR similar to breakage of fragile sites at inverted repeats that has been proposed to occur due to replication problems [35], [46], [47] in contrast to mechanical breakage [55], [56] of the initial dicentric GCR chromosomes during mitosis when the two centromeres are pulled in opposite directions. In one GCR of this class analyzed, one of the resulting broken DNA ends appeared to be involved in a subsequent BIR event with homologous sequences located on the same chromosome arm of the intact copy of the initial target chromosome in the inverted orientation. In the other GCR of this class analyzed, the broken end of the centromere containing fragment of the initial dicentric translocation appeared to be involved in a subsequent BIR event with a repeated sequence on itself associated with template switching resulting in a second dicentric GCR. Subsequent multiple BFB cycles associated with BIR and template switching targeting the intact copy of the initial target chromosome could account for the observed sequence amplifications and the final GCR structure.

In the third class of dicentric resolution events, resulted in translocation or interstitial deletion with the second breakpoint at either a region of microhomology or non-homology which were not different from primary GCRs breakpoints found at regions of microhomology or non-homology [30]. In one example a dicentric GCR was found to be stabilized by interstitial deletion of a region containing one of the centromere sequences where the deletion breakpoint was at a region of non-homology. This is similar to previous observations that genetically engineered dicentric chromosomes can break at or near the centromere followed by end resection and rejoining by NHEJ to delete a centromere [39], [40] indicating that both engineered dicentric chromosomes and spontaneous dicentric GCRs can be resolved by the same mechanism. It was also previously observed that centromere deletion could occur by breakage of an engineered dicentric chromosome followed by healing of the free ends by telomere addition [39]. However, this second pathway, which requires a functional telomerase [27], [58], was not observed in our studies probably because most of the dicentric GCRs characterized in our structural studies were isolated in *tlc1* mutant strains.

In the fourth class of dicentric resolution, the second breakpoint was at a region of microhomology or non-homology fused to a telomere, similar to previously characterized chromosome fusion junctions [27], [30]. These fusions were observed in telomerase defective strains and might be facilitated by the loss of telomere protection in these mutants. Fusions to the right arm of chromosome V created circular chromosomes, whereas fusions to other chromosomes generated a new dicentric chromosome. New dicentric chromosomes would be predicted to initiate an additional BFB cycle and lead to additional rearrangements, although we did not study these GCRs further to verify this.

### HR, NHEJ and Telomerase can influence the resolution of dicentric GCRs

Previous analysis of more than 350 sequences at the breakpoints of translocations, isoduplications and chromosome fusions targeting the left arm of chromosome V revealed that most monocentric and dicentric rearrangements were likely formed by NHEJ or by some type of recombination at very short repeated sequences [30], although it should be noted that the chromosome V breakpoint region targeted by these rearrangements does not contain repeated sequences other than the *CAN1* gene that shows divergent homology with several other genes on different chromosomes [59]. Out of 13 resolved dicentric GCRs studied here, the resolution of 9 dicentric GCRs involved rearrangements targeting genomic repeat sequences suggesting that secondary rearrangements were mediated by HR, and the resolution of the remaining 4 dicentric GCRs involved non-homology or microhomology breakpoints. Consistent with this bias toward HR mediated secondary breakpoints, we observed a significantly decreased frequency of predicted dicentric GCRs in HR-deficient strains compared to that seen in other mutant backgrounds. Taken together, these observations suggest that as long as repeated sequences are present in secondary breakpoint region and independently of the mechanism leading to the initial dicentric GCR, HR is likely the most efficient mechanism resulting in secondary rearrangements. In contrast, in the absence of repeated sequences in the secondary breakpoint region, other mechanisms including NHEJ can result in secondary rearrangements.

As expected, predicted dicentric GCRs are more often observed in telomerase deficient strains compare to telomerase proficient strains. The chromosome fusion class of dicentric GCRs, formed by the fusion of a broken chromosome V to different telomeres [15], [27], [28], [31], are almost exclusively observed in telomerase defective strains, suggesting that abnormal telomere structures, such as those produced by recombinational maintenance of telomeres, are likely required for such fusions to occur [27], [28]. There are other additional explanations for how the formation and recovery of dicentric GCRs may be more frequent in telomerase deficient strains. First, *de novo* telomere addition is a potent mechanism for healing broken chromosomes [15], [27], [29], [60], [61] and eliminating this pathway with telomerase defects would likely channel broken chromosomes into other pathways, some of which could yield dicentric GCRs [16], [27], [28]. Second, we observed Ty element and delta sequences at many secondary rearrangement breakpoints in telomerase defective strains [30], raising the possibility that the activation of transposons in telomerase deficient strains [62] might affect the efficiency of HR mediated secondary rearrangements at Ty elements and delta sequences. Finally, it is possible that checkpoint activation in post-senescent telomerase deficient strains [63], even in *mec1* mutants [64], could facilitate HR and hence contribute to promoting the resolution of dicentric GCRs by HR.

### Propagation of chromosomes lacking telomeres

We observed that in all *mec1 sml1 tlc1* and *mec1 sml1 lig4 tlc1* post-senescence strains analyzed, telomeres were shorter than in wild-type strains and often telomeres were absent and a variable amount of DNA was lost at one to three chromosomes ends in the population of cells from which the DNA was isolated. From these observations we suggest that although telomere sequences are maintained by HR, in *mec1 sml1 tlc1* mutant strains some telomeres are not efficiently maintained and are shorter than normal, which is consistent with a reduced HR efficiency in *mec1* mutants [64]. In some cases the telomeres are lost and the resulting chromosome ends continually shorten as the mutants continue to divide because these unprotected ends cannot induce checkpoint-dependent cell cycle arrest in the absence of Mec1. The rate of shortening appears to be slow enough that the cells can go through a considerable number of cell divisions before essential genes are lost and the cells can ultimately no longer divide. The chromosome end decay observed in these mutants appears to be different from the chromosome end decay observed in *exo1 rad52 tlc1* survivors, which terminates due to chromosome end protection and amplification by palindrome formation at small inverted repeats resulting in chromosomes that can be stably maintained [32]. Our aCGH analysis did not reveal DNA amplification at a defined, shortened chromosome ends characteristic of this type of survivor but rather revealed a population of heterogeneous shortened chromosomes ends suggestive of continuous shortening supporting the view that aberrant chromosomes without telomeres can propagate for a considerable period of time when checkpoint functions are defective. Degrading ends can be stabilized by fusion to another broken chromosome end or to an unprotected telomere contributing to the formation of primary dicentric GCRs. In checkpoint deficient strains extensive end degradation up to and past a centromere could provide for an alternative mechanism to initiate secondary rearrangements of dicentric GCRs in addition to other breakage mechanisms including mechanical breakage or inverted repeat-induced DSBs.

### Conclusion

**Figure 8:**
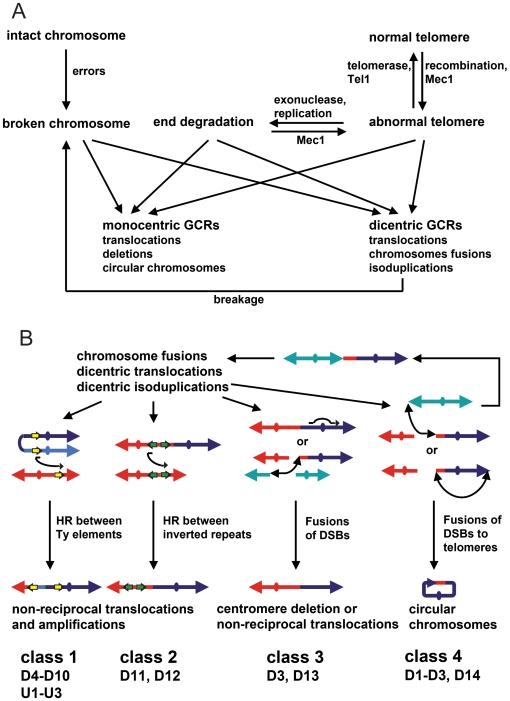
Formation and resolution of dicentric GCRs. (A) DNA damage resulting from replication errors, activation of fragile sites, untimely replication, defects in chromatin assembly and oxidative stress as well as defects in the S-phase and DNA damage checkpoints appear to result in broken chromosomes. In the absence of telomerase, telomeres are maintained by HR; however when HR is partially compromised or when other pathways that suppress GCRs are compromised, such telomeres are prone to fuse to broken DNAs and other telomeres. In addition Mec1 appears to facilitate the efficient maintenance of telomeres by HR and in the absence of Mec1 such telomeres can be degraded resulting in the formation of terminally deleted chromosomes. These aberrant chromosomes can then participate in a diversity of genome rearrangements. (B) Illustration of the four classes of resolution events observed that further rearrange dicentric GCRs until a stable, monocentric GCR can be formed. Of the 19 potential dicentric GCRs analyzed, it was possible to assign 17 GCRs to specific classes of secondary rearrangements even though not all of the GCRs were completely analyzed. The remaining 2 GCRs were not classified because they were not analyzed to a sufficient level of detail.

Previous studies have demonstrated that a diversity of genetic defects as well as treatment with DNA damaging agents can result in broken chromosomes leading to the formation of GCRs [18], [24], [58], [65] (**Figure 8A**). The results presented here are consistent with a model where dicentric GCRs are a source of broken chromosomes that participate in secondary rearrangements resulting in monocentric GCRs that can be stably transmitted during cell division and this study present for the first time a comprehensive overview of the mechanisms contributing to the resolution of such dicentric GCRs (**Figure 8B**). Stabilization of broken dicentric GCR chromosomes by HR is highly efficient and often appears to be mediated by BIR events between transposon elements that are distributed genome wide. In addition, if inverted repeat sequences are present at secondary breakpoint junctions, these sequences can be amplified, possibly through multiple rounds of BIR and/or template switching during BIR. The healing of the broken dicentric GCRs can also occur by NHEJ resulting in interstitial deletion of a centromere, non-reciprocal translocations and chromosome fusions similar to the events resulting in primary GCRs. The resolution of dicentric GCRs can be a multistep process, as some resolution events can result in the formation of secondary dicentric GCRs which continue to be unstable resulting in additional BFB cycles. Overall we have established that in haploid *S. cerevisiae* multiple pathways contribute to the protection of telomeric and non-telomeric chromosome ends and incorrect repair producing dicentric GCRs can ultimately result in multiple karyotypic alterations including genes amplification between genomic repeats and deletion of non-essential chromosomes fragments potentially resulting in a enormous diversity of complex GCRs. Similar mechanism have been proposed to contribute to the formation amplifications and non-reciprocal translocations initiated by dicentric chromosomes formed by sister chromatid fusions or telomere fusions in mamalian cells [3], [8], [10]–[12].

## Materials and Methods

### Strains

*S. cerevisiae* strains used in this study are isogenic to RDKY3615 (*MAT* **a** *ura3-52 trp1Δ63 his3Δ200 leu2Δ1 lys2ΔBgl hom3-10 ade2Δ1 ade8 hxt13*::*URA3*). All strains were constructed by intercrossing and three separate *MAT* **a** isolates were selected. The post-senescence *tlc1* mutants survivors were generated by subculturing the selected clones in liquid media. The post-senescence survivors were genotyped by PCR and the survivor types were determined by southern blotting of Xho I-digested genomic DNA with a poly(CA_1–3_/TG_1–3_) probe. A total of 15 single colonies (5 for each isolate) were used to inoculate liquid cultures in non-selective media and plated on plates containing canavinine and 5-fluoroorotic acid. Single Can^r^ 5FOA^r^ isolates arising in seperate cultures to avoid obtaining multiple isolates of individual rearrangement events were previously described [28] and the GCR breakpoint sequences from the GCRs present in these isolates are presented in **Figure S1**. Of the 21 Can^r^ 5FOA^r^ isolates selected for further analysis as described in this study, a number of them were isolated from the same Can^s^ 5FOA^s^ parental strain including the D2 and D5 GCRs, the D4 and U1 GCRs, the D6, D7 and D8 GCRs, the D9 and U2 GCRs, the D10 and D15 GCRs and the D13 and D16 GCRs.

### Pulsed Field Gel Electrophoresis (PFGE)

The Can^r^ 5FOA^r^ isolates were grown to log phase in 100 ml of YPD at 30°C. Prior to sample preparation, portions of the cultures were reserved for genomic DNA extraction. The remainder of the cells were washed three times in 10 ml of ice cold 10 mM Tris pH 7.5, 50 mM EDTA pH 8 and resuspended in 110 µl of 10 mM Tris pH 7.5, 500 mM EDTA pH 8 containing 2.5 mg/ml zymolyase-100T (ICN). The cells were then pre-warmed briefly at 37°C and mixed 1∶1 with warmed (42°C) liquefied 1.2% Incert Agarose (Biowhittaker Molecular Applications) in 125 mM EDTA pH 8 to prepare multiple 80 µl plugs containing 2×10^8^ cells per plug. The plugs were incubated in 500 µl of 10 mM Tris pH 7.5, 500 mM EDTA pH 8, 1 mg/ml zymolyase-100T, 1% 2-Mercaptoethanol for 24 h at 37°C. The plugs were then rinsed 30 min in 10 ml of 10 mM Tris pH 9.5, 500 mM EDTA pH 8, 1% Sodium N-Lauryl Sarcosine, 0.2% Sodium Dodecyl Sulfate at room temperature in 15 ml conical tubes and then incubated in 500 µl 10 mM Tris pH 9.5, 500 mM EDTA pH 8, 1% Sodium N-Lauryl Sarcosine, 0.2% Sodium Dodecyl Sulfate containing 2 mg/ml Proteinase K (EM Scientific) for 48 h at 55°C. To digest the DNA with *Asc* I (New England Biolabs) prior to PFGE, plugs were extensively washed in digestion buffer and incubated with 60 units of *Asc* I in 500 µl of 1X digestion buffer at 37C for 18 h. Finally, the plugs were extensively washed in 10 mM Tris pH 7.5, 50 mM EDTA pH 8 prior to resolving the chromosomes in a 1% Agarose gel run in a CHEF (clamped homogeneous electric field electrophoresis) apparatus in chilled (4°C) 0.5X TBE (89 mM Tris-borate, pH 8.3, 25 mM EDTA) under two different conditions; 7 V/cm with 75 sec or 90 sec fixed pulse times for 24 h. Gels were stained with 0.5 µg/ml ethidium bromide for 30 minutes to visualize the chromosomes.

### Southern blotting

To determine telomere sizes, purified chromosomal DNA from each strain was digested with *Xho* I (New England Biolabs) and separated by electrophoresis through a 0.7% agarose gel. In the case of chromosomes resolved by PFGE, the DNA was fragmented by UV-irradiating the gel in a Stratalinker^TM^ (Stratagene) apparatus at maximum output for 60 seconds. Following neutral capillary transfer of the DNA from the gels onto nitrocellulose membranes, a ^32^P-labeled probe made by a random priming was hybridized to the DNA on the membranes. After overnight of hybridization in QuikHyb hybridization solution (Stratagene), the nitrocellulose filter was washed stringently and the radioactivity was detected using a PhosphoImager (Molecular Dynamics, Inc.). Southern Blot probes used were the telomeric TG repeat fragment obtained from pBC6 [66] or chromosome V [43162-43877] and chromosome XIV [95390-95949] fragments that had been previously amplified by PCR, cloned into pCR2.1-TOPO vector (Invitrogen) and sequenced.

### Array Comparative Genome Hybridization analysis

Genomic DNA from the RDKY3615 Can^s^ 5FOA^s^ strain and from each Can^r^ 5FOA^r^ GCR strain was purified using a Gentra Puregene Yeast Kit (Qiagen) following the protocol provided by the manufacturer. The DNA was fragmented and labeled with either Cy3 or Cy5. Then Cy3-labeled DNA from the reference strain RDKY3615 was mixed with an equal amount of Cy5-labeled DNA from a given individual isolate containing a GCR and then hybridized to a high density microarray containing 50 bp sense and anti-sense oligonucleotides spaced by 15 bases across the *S. cerevisiae* genome. All DNA labeling and hybridization to microarrays was performed by NimbleGen. The log_2_ ratio of the fluorescence intensities for each oligonucleotide and a log_2_ ratio value less than 0.5 relative to the reference signal was interpreted as indicating a region of deletion in the GCR strain whereas a log_2_ value above 0.5 relative to the reference signal was interpreted as indicating a region of amplification in the GCR strain. The aCGH data is available at http://www.ebi.ac.uk/arrayexpress accession number E-TABM-732.

### Quantitative PCR

Amplifications and deletions identified by aCGH were verified by qPCR. qPCR was performed on a real time PCR detection system from BioRad using SYBR green quantitative PCR mix (BioRad) according to the manufacturer's recommendations. Primers were selected based the region of interest identified by aCGH analysis. Reactions were performed in triplicate with both test and control genomic DNA. Relative amounts were calculated using the ΔCT method. Nucleotide sequences of the primers used for the qPCR reactions are available upon request.

## Supporting Information

Table S1 (0.04 MB PDF)

Figure S1 (2.94 MB PDF)
