# Supplementary material for: Stabilization of Dicentric Translocations through Secondary Rearrangements Mediated by Multiple Mechanisms in S. cerevisiae
Source: PLoS One. 2009 Jul 28;4(7):e6389. doi: 10.1371/journal.pone.0006389 (PMC2712687; doi:10.1371/journal.pone.0006389)
Supplement: Table S1 — (0.04 MB PDF) [file pone.0006389.s001.pdf]

Table S1: Distribution of dicentrtric GCRs recovered from different strain backgrounds

Strain background	Translocation class	telomerase proficient	telomerase defective [e]
wild-type	non-reciprocal translocation	3	1
	dicentric isoduplication	1	1
	chromosome fusion		16
checkpoint defective [a]	non-reciprocal translocation	1	6
	dicentric isoduplication	1	1
	chromosome fusion	1	22
HR defective [b]	non-reciprocal translocation	1	2
	dicentric isoduplication	1	3
	chromosome fusion		20
NHEJ defective [c]	non-reciprocal translocation	2	1
	dicentric isoduplication	2	
	chromosome fusion	1	
checkpoint & HR defective	non-reciprocal translocation		4
	dicentric isoduplication		1
	chromosome fusion		10
checkpoint & NHEJ defective	non-reciprocal translocation		3
	dicentric isoduplication		7
	chromosome fusion		5
other [d]	non-reciprocal translocation		2
	dicentric isoduplication	4	3
	chromosome fusion	1	14
TOTAL	non-reciprocal translocation	7	19
	dicentric isoduplication	9	16
	chromosome fusion	3	87

[a] checkpoint deficient strains includes strains that contain *chk1*, *dun1*, *mec1*, *mec3*, *pds1*, *rad9*, *rad53* and/or *tel1* mutations

[b] HR deficient strains includes strains that contain *rad51*, *rad52*, *rad54*, *rad55*, *rad59* and/or *rdh54* mutations

[c] NHEJ deficient strains includes strains that contain *lig4*, *ku70*, *ku80* or *mre11* mutations

[d] Other deficicnet strains includes strains that contain *asf1*, *exo1*, *rfc5-1* or *top3* mutations

[e] Telomerase deficient includes all strains that contain *tlc1* or *est2* mutations
