# Supplementary material for: Stabilization of Dicentric Translocations through Secondary Rearrangements Mediated by Multiple Mechanisms in S. cerevisiae
Source: PLoS One. 2009 Jul 28;4(7):e6389. doi: 10.1371/journal.pone.0006389 (PMC2712687; doi:10.1371/journal.pone.0006389)
Supplement: Figure S1 — (2.94 MB PDF) [file pone.0006389.s002.pdf]

M1 : *rad51 tlc1* mut 23

breakpoint sequence

monocentric translocation

GTTGGGTTTCCTCTTTGATTAACGCTGCCTTCACATTTCAAGGTACTGAACT:AGTTGGTATCACTGCTGGTGAAGCTGCAAACC chr V 32562

GATGGGTTTCCTCTTTGATTAATGCTGCATTTACGTACCAAGGTACTGAACT:GGTTGGGATAACCGCTGGTGAAGCGGCTAACC chr XIV 136809

aCGH analysis

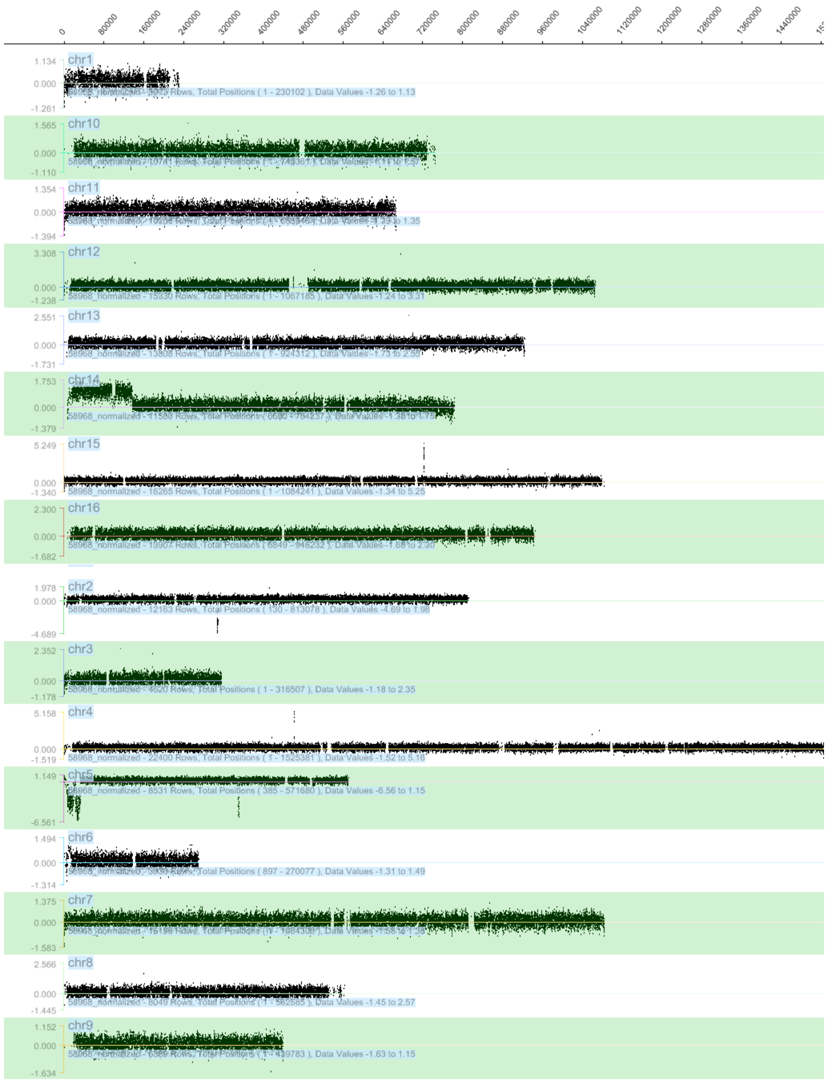

Observed karyotype modifications

del5[1-32600] telomere to *CAN1*
dup6[1-13400] L telomere
dup14[1-136720] telomere to *ALP1*

Observed GCR size : 679 Kb

Calculated GCR size : 679 Kb

M2 : *rad55* mut 3

breakpoint sequence

de novo telomere addition

GATGGTCTTTCTGGTTTCAAGTTGCCGGATTGTGCCGTATTTCTAGACCCTGTGG:gtgtgggtgtgggtgtgggtgtgggtgt [34989]

aCGH analysis

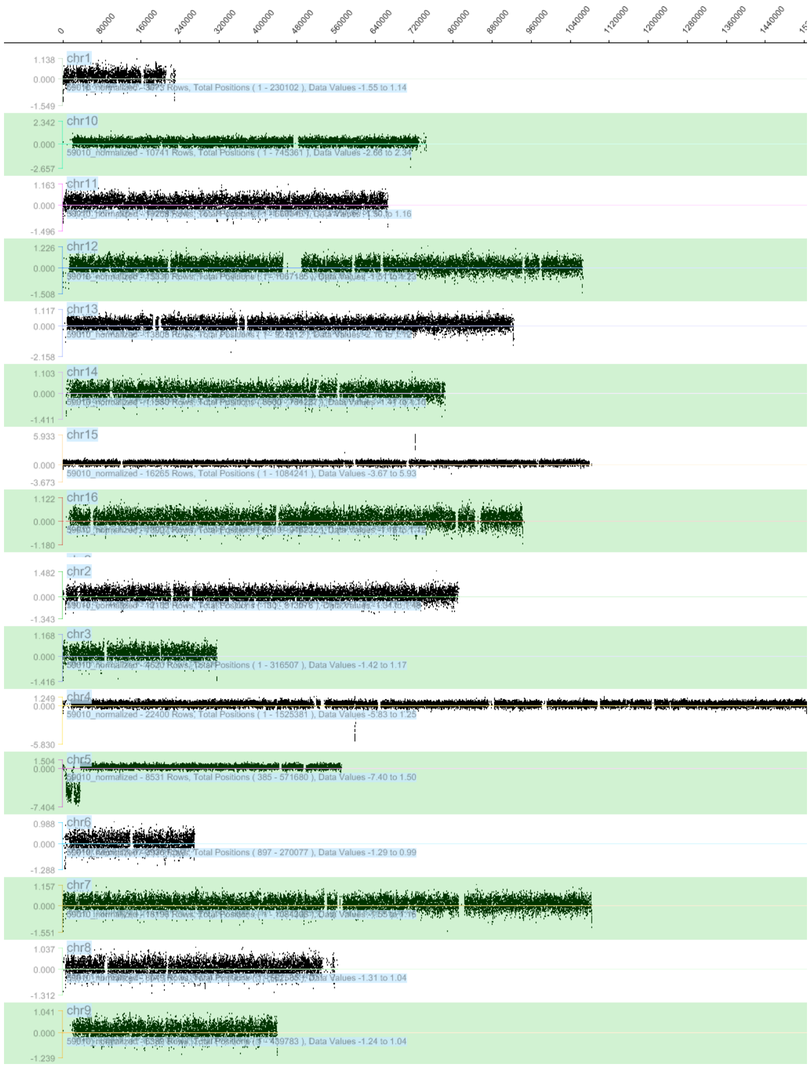

Observed karyotype modifications

del5[1-34989]

Observed GCR size : 539 Kb Kb

Calculated GCR size : 539 Kb

D1 : *tel1 tlc1* mut 6

breakpoint sequence

chromosome fusion to the Y′ element of an unidentified telomere

GAAGTGGCGGGCCCATTATTCCAGGATTTCATTAAAG:GTTACAATTGCACCGTACTGGTATATGG chr V 39183

AAAAGTACTTTATCTCAATGGGTTTACGTGGGCATAG:ATCACGCTTCAGCCGCTCTGTGTCGACT chr unidentified

aCGH analysis

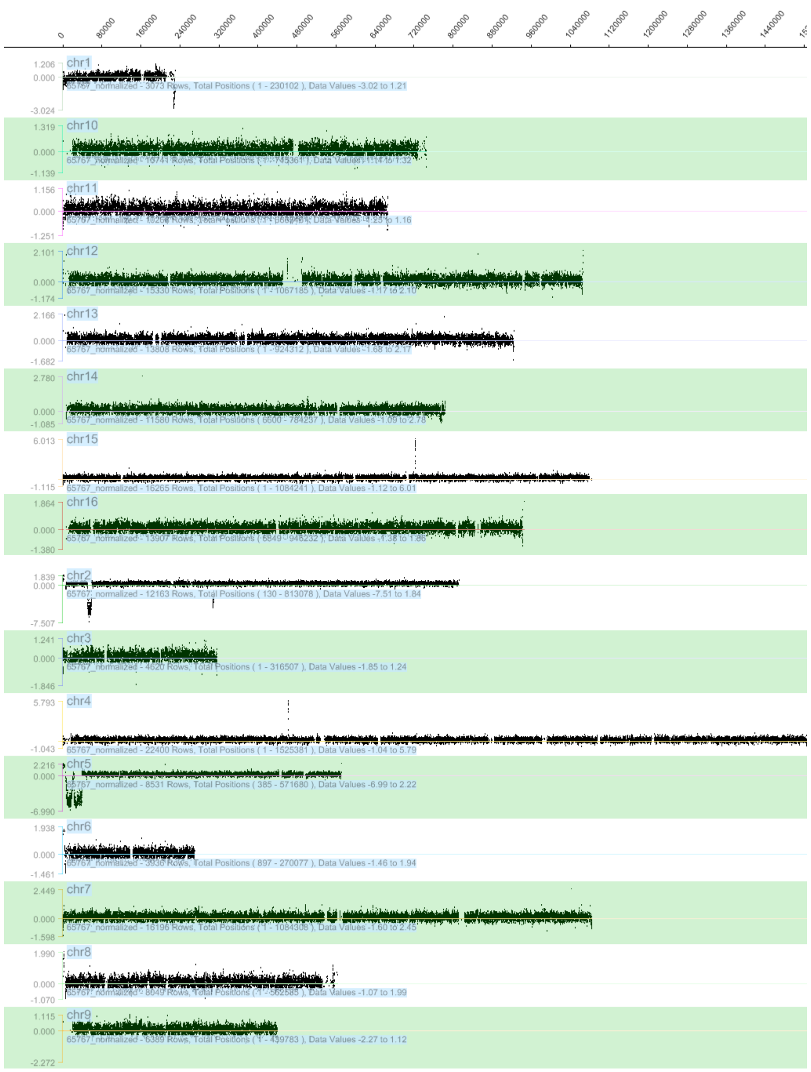

Observed karyotype modifications

dup1[190554-192218]
del1[226800-230208]
dup2[1-1986]
del2[1986-7106]
del3[300-1218]
del5[1-39183]
dup6[1-4800]
dup8[1-4000]
del11[1-1797]
del11[664490-666217]
dup12[1065840-1078174]

Observed GCR size : 575 Kb

Calculated GCR size : 535 Kb

D2 : *mec1 sml1 tlc1* mut 14

breakpoint sequence

chromosome fusion to an unidentified telomere
GCCGAAGACGCTTTCTTCATTTCTACTAAAGACACC:TCCTTGTCCACCTCTGAGGCTGGCGGTATCCAGGA chr V 41273
CGTCAGAAAGACAAACTTCTCCCAAAATGTATTACC:CCGCCGAATAAGAAAGCAGACCCATTCACCAACGA chr unidentified

aCGH analysis

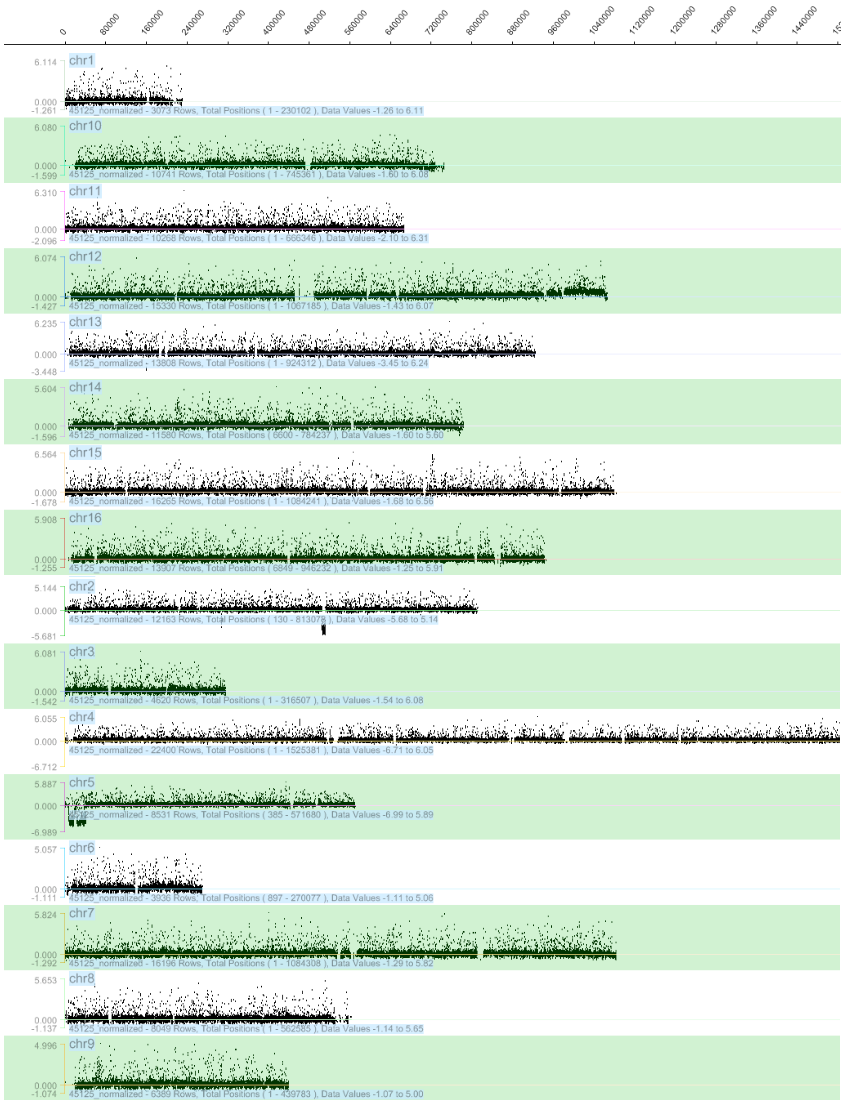

Observed karyotype modifications

del5[1-41316] L arm
dup12[947121-976250] delta 22 to delta 25
trip12[981692-1078174] delta 25 to telomere
chromosome end decay 7[1076841-1090947]
chromosome end decay 10[706841-74541]

Observed GCR size : 575 Kb

Calculated GCR size : 535 Kb

D3 : *rad55 tlc1* mut 14

breakpoint sequence

```
                first breakpoint              second breakpoint

chr V  [33901]  ACCAAGGTCAATAAT:ATATCTTTTAGTATA [33872]
chr XI [430506] CGTACACACGAAGAT:CCTTTCTGAGTAGCG   //   TGTTCAAGGAAAATA:TCAGATAAAAAATTT [430694]
                                      chr XI [429875] TCTTCATTCTATATA:CCCTGTTTAAGTTAT [429846]
```

aCGH analysis

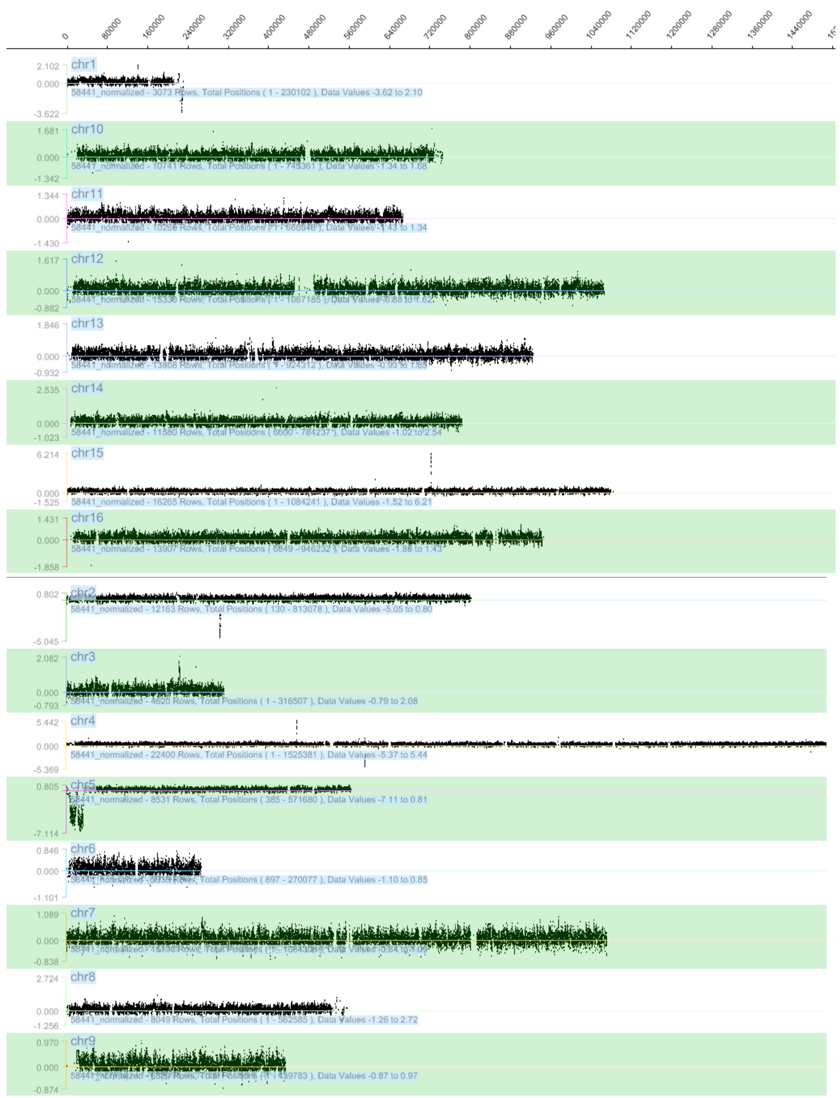

Observed karyotype modifications

del1[222558-230208] *YAR068* to R telomere
del5[1-34008] L arm
dup7[270836-273657] *SCS3* and *MET13*
dup10[462524-472699] *YJR014* to delta 11

Observed GCR size : 540 Kb

Calculated GCR size : 540 Kb

D4 : *mec1 sml1 tlc1* mut 3

breakpoint sequence

dicentric isoduplication
GAGAAAGAAAGTCGCTTCAAGCTAACCCAGTTTTCT:TTTTTTTTATCACTTATCAGTCCTATTCGGAGATA chrV [34333]
AGGCAATATCAACTATTCAATCTTTAACATTTTTCT:CGAGCCCTCATTGTCCACATTTTCACTGGATTTGT chrV [36558]

aCGH analysis

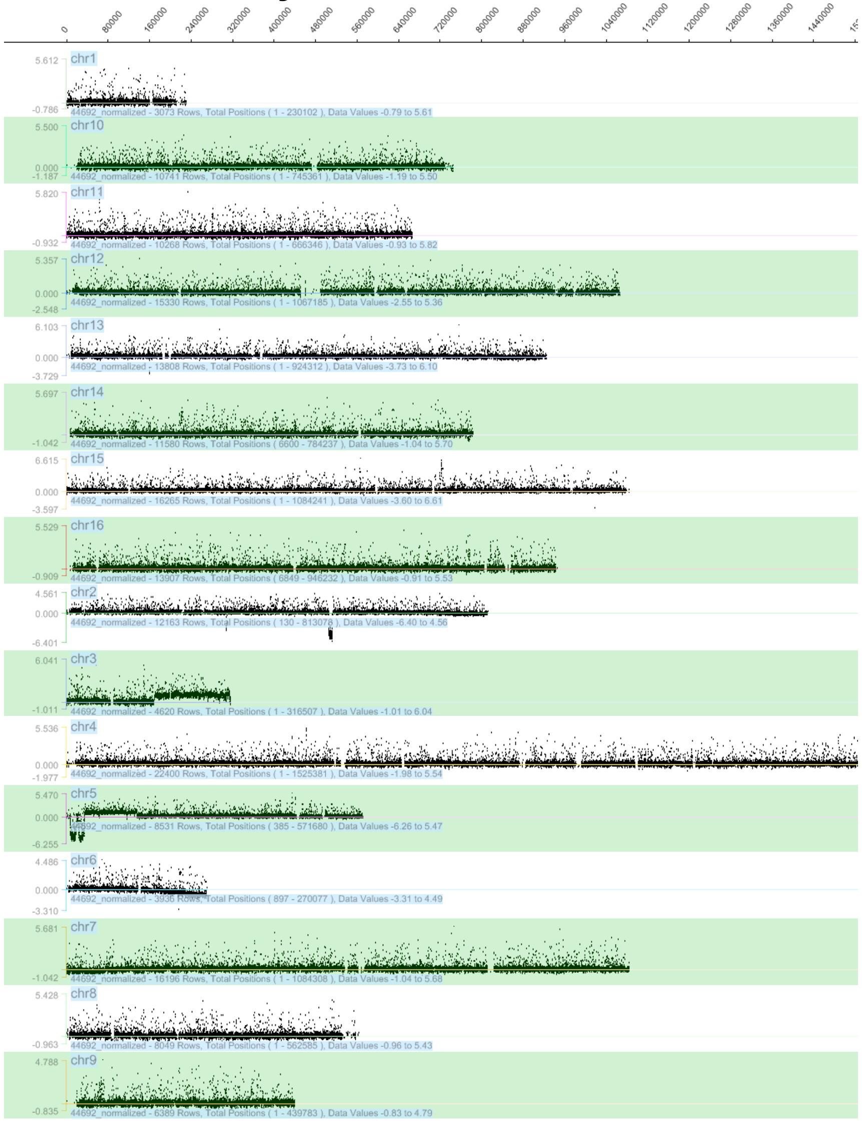

Observed karyotype modifications

chromosome end decay 2[690232-813178]
dup3 [169569-316617] delta 11 to the telomere
del5 [1-34333] L arm
dup5[36558-135612] first BP to delta 4
chromosome end decay 6[148501-270148]
dup11[63850-64362] YKL202
chromosome end decay 13[833439-924429]

Observed GCR size : 740 Kb

Calculated GCR size : 789 Kb

D5 : *mec1 sml1 tlc1* mut 15

breakpoint sequence

dicentric isoduplication
CCCTTATTAG**C**CTTGATAGTGCTG**AAAAAAAGAAAA:**AAAACAAAAAAAAAG**A**AAT**A**AAA**T**AACGGCAAA**AC**AG chr V [34101]
CCGAATAGGA**C**TGATAAGTGATAA**AAAAAAAGAAAA:**CTGGGTTAGCTTGA**A**GCGA**C**TTT**T**CTTTCTCT**AC**TA chr V [34324]

aCGH analysis

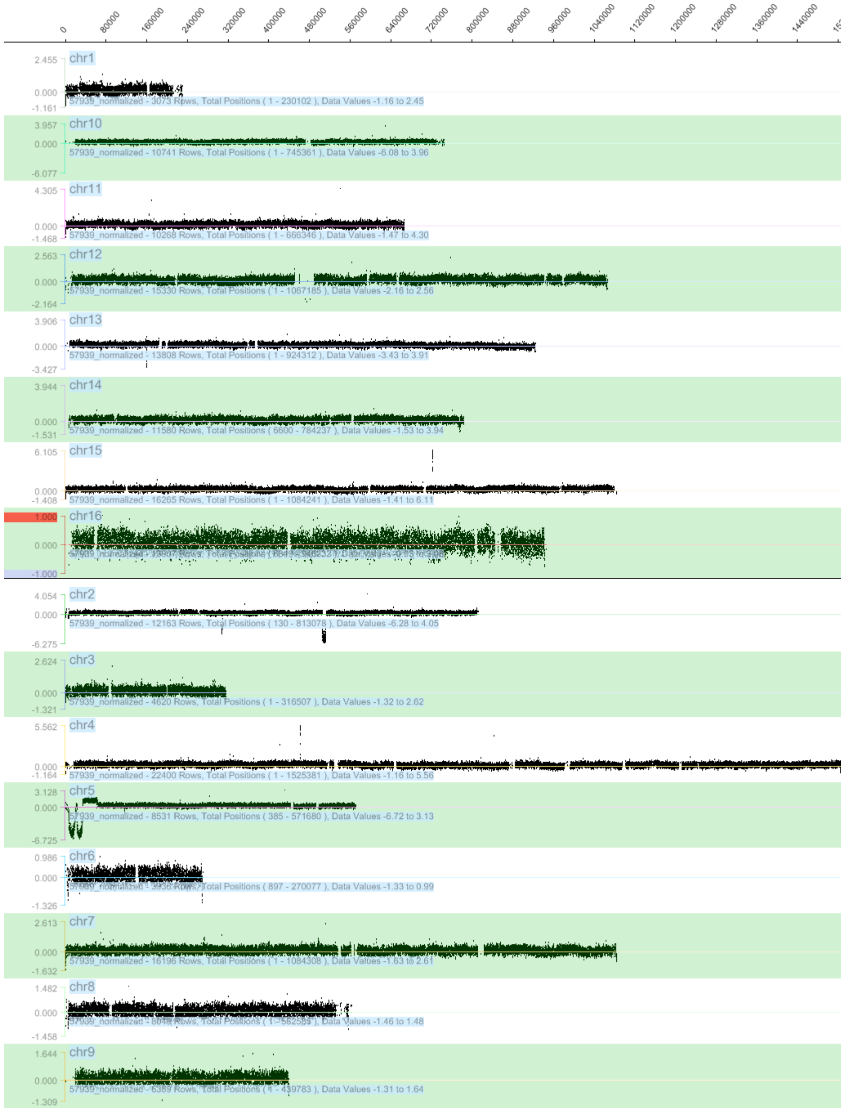

Observed karyotype modifications

del1[1-12000] L telomere
dup1[209439-230208] R telomere
del2[1-7000] L telomere
del5[1-34101] L arm
dup5[34324-63728] first BP to delta 1
del9[438216-439885] *YIR044* to telomere

Observed GCR size : 580 Kb

Calculated GCR size : 593 Kb

D6 : *mec1 sml1 lig4 tlc1* mut 21

breakpoint sequence

dicentric isoduplication

GAGACTCTATTGTCGAGGCTACAGAATTTGACACTC:AAAATAGCGCCCCATGGGGGTTGGCCCGTATTTCCC chr V [41101]

CGAGCAGACGTATTCGGCTCCTTTGAATTGGCACTC:CCATTGCACAAGCTCTTTCAGCCGGCAGTTGGTTTG chr V [42523]

aCGH analysis

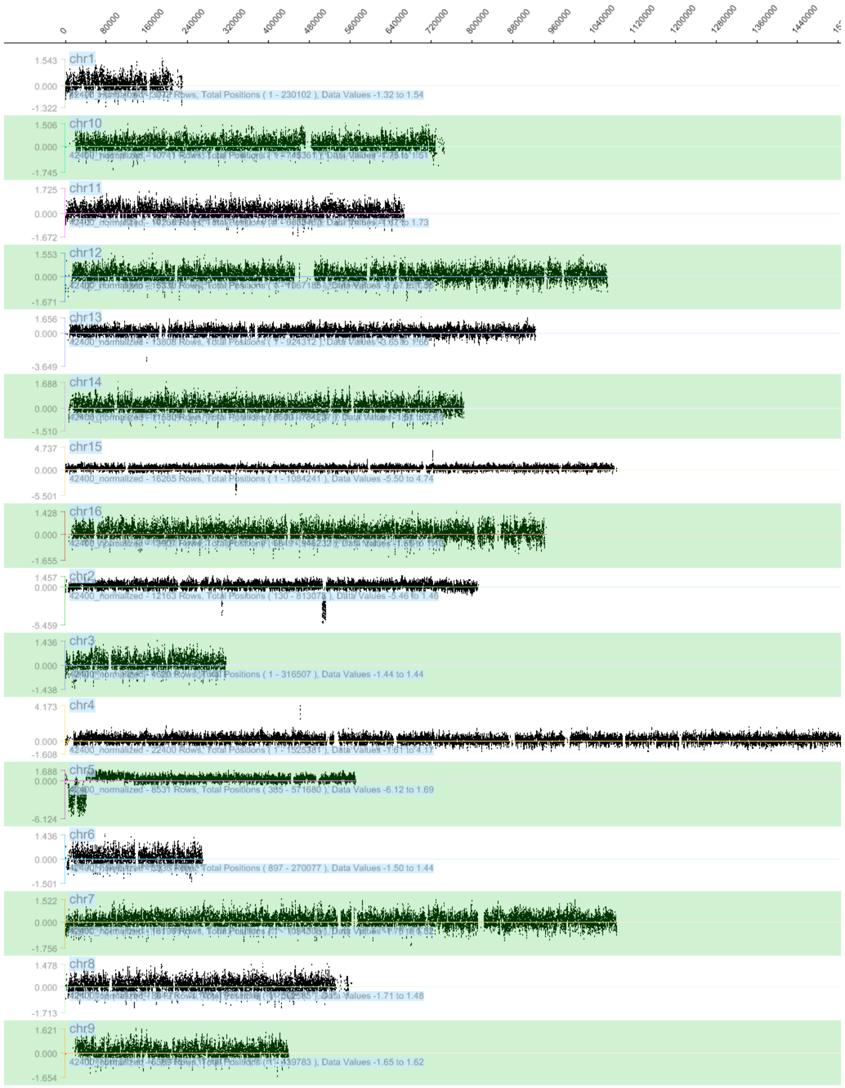

Observed karyotype modifications

del5[1-41101] L arm

dup5[42523-135612] second BP to delta5

chromosome end decay 7[1-38745]

dup10[462460-472699] from *YJR008* to delta 11

Observed GCR size : 1,300 Kb

Calculated GCR size : 630 Kb

D7 : *mec1 sml1 lig4 tlc1* mut 22

breakpoint sequence

dicentric isoduplication

GTGAAATGGCTACATTCATCCCTGTTACATCCTCTT:TCACAGTTTTCTCACAAAGATTCCTTTCTCCAGCAT chr V 33010

AGAAAAAGAAAAAAAAAAAGCAGCTGAAATTTTTCTA:AATGAAGAATTAAATAATATTCAATTTATCAAGAAT chr V 40036

aCGH analysis

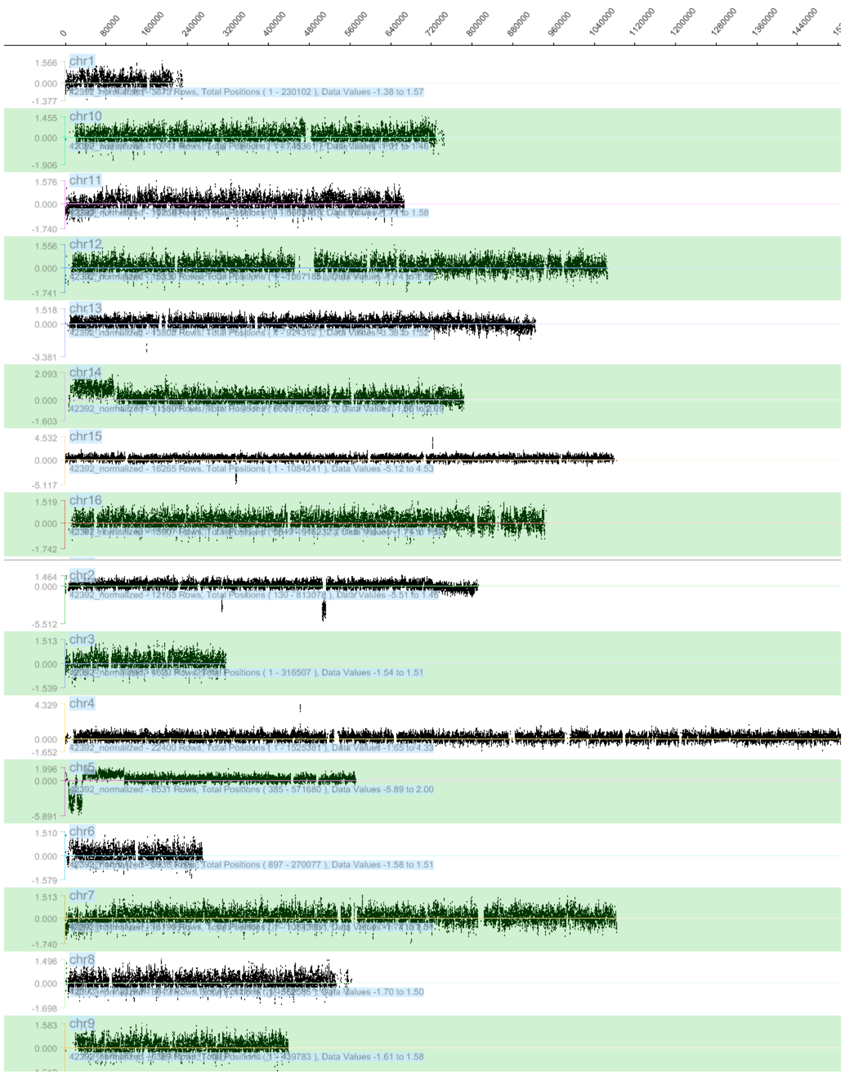

Observed karyotype modifications

dup1[190234-192282]
chromosome end decay 2[723349-813178]
del5[1-33944] L arm
dup5[40036-116167] second BP to *URA3-52*
chromosome end decay 7[1-91452]
dup10[462460-472099] from *YJR008* to delta 11
chromosome end decay 11[1-57066]
chromosome end decay 13[864183-924429]
dup14[1-102523] L telomere to delta 1

Observed GCR size : 745 Kb

Calculated GCR size : 712 Kb

D8 : *mec1 sml1 lig4 tlc1* mut 24

breakpoint sequence

```
dicentric isoduplication
```

TCC**A**CA**A**GG**AA**AACGTCC**A**GCA**GGCACAACTTCAAT:**CCGT**A**GAG**A**ACTTATCTGCCGAAGACGCT**TTCT**TCA chr V [41326]

GAT**A**TT**A**TT**AA**TGATAAT**A**AAT**GGCACAACTTCAAT:**AAAG**A**TCT**A**TTCGGCACTGTTTCAGAAGA**TTCT**CTC chr V [47828]

aCGH analysis

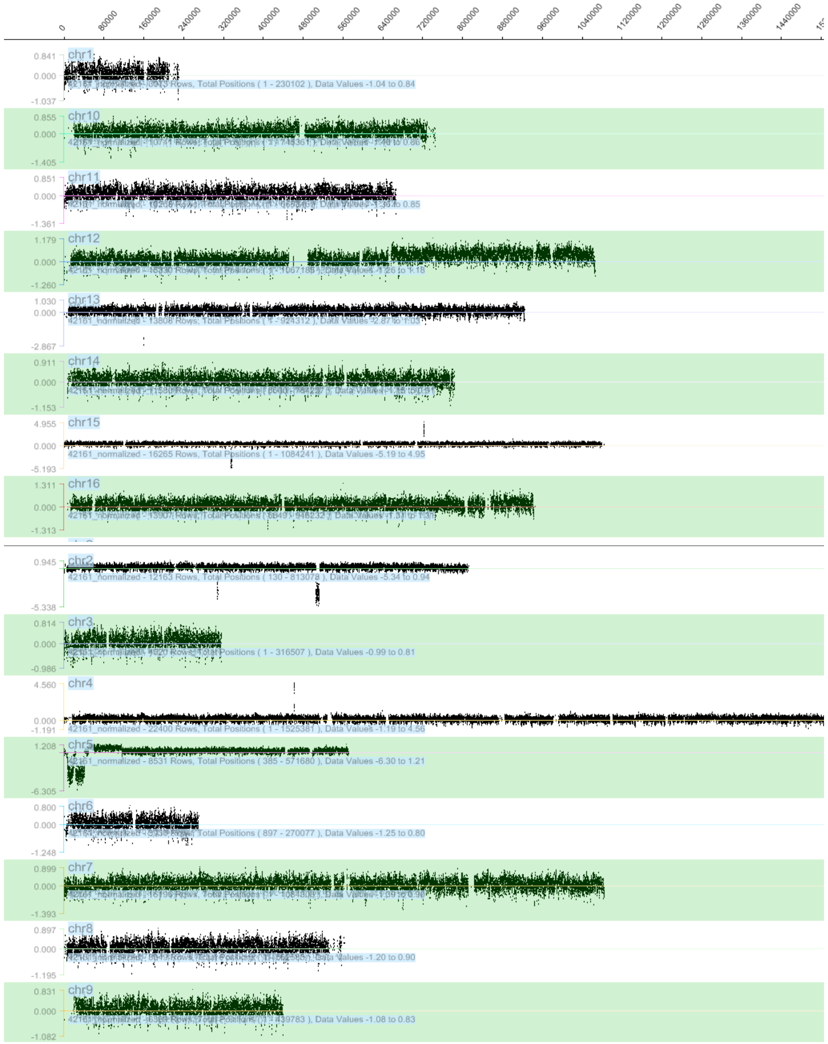

Observed karyotype modifications

del5[1-41326] L arm
dup5[47828-116167] second BP to *URA3-52*
del12[670966-672566] *RED1*
dup12[593147-1078175] delta 10 to R telomere
del16[408339-408979] *ATP4*

Observed GCR size : 1,080 Kb

Calculated GCR size : 1,088 Kb

D9 : *mec1 sml1 lig4 tlc1* mut 26

breakpoint sequence

dicentric isoduplication
GTTGAAGTCGGTATTCACTTTGCCGTGGCTGCTGGTA:ACGAAAACCAAGACGCTTGTAATACCTCCCCAGCT chr V [40594]
AGTCATCATTCCTCAAACCTCCTTGCGGCCGCAGGTA:TGGACTCGTATGTTTATCTATATGATCTAAGAAAC chr V [47982]

aCGH analysis

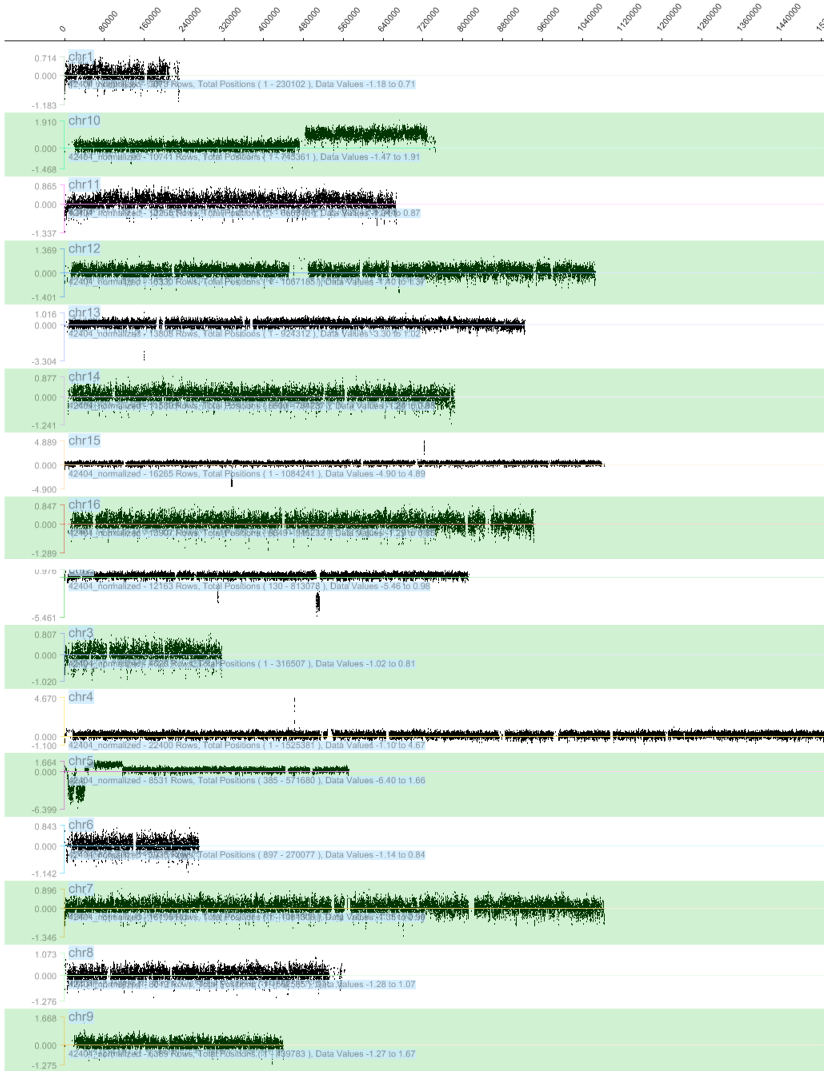

Observed karyotype modifications

del5[1-40594] L arm
dup5[47982-1160167] second BP to *URA3-52*
dup10[472455-745741] delta 10 to R telomere
chromosome end decay 11[1-10194]
chromosome end decay 11[658151-666454]
del12[671286-673145] *RED1*

Observed GCR size : 860 Kb

Calculated GCR size : 877 Kb

D10 : *mec1 sml1 lig4 tlc1* mut 34

breakpoint sequence

dicentric isoduplication

ATAGGTGATGAAGATGAAGGAGAAGTACAGAACGCTG:AAGTGAAGAGAGAGCTTAAGCAAAGACATATTGGT chr V [33220]

AGAATATTACGTTTTCAAGATGACGAACAGAACGCTG:GTGAAGACGATAACGATATAATGTCACCGCTTCCT chr V [89383]

aCGH analysis

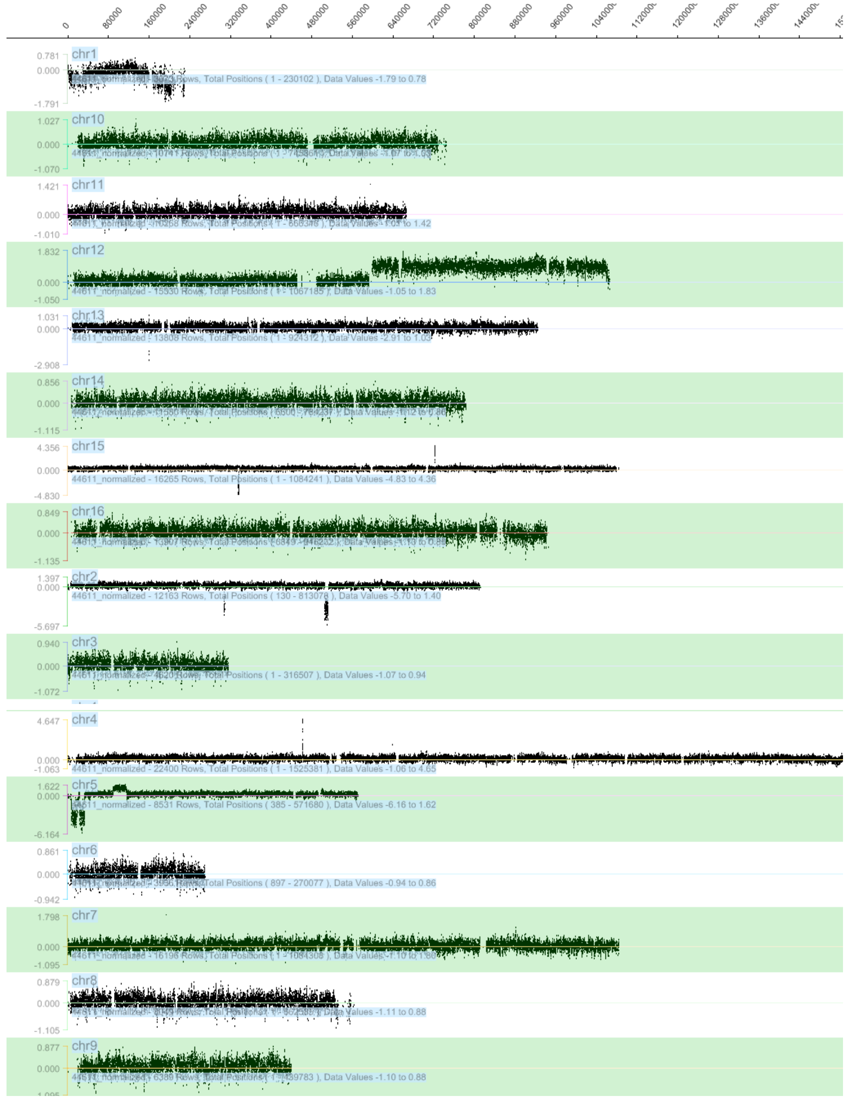

Observed karyotype modifications

chromosome end decay 1[1-74434]
chromosome end decay 1[135744-230208]
del5[1-33220] L arm
dup5[89383-116167] second BP tp *URA3-52*
del10[29761-33217] *YJL21*
dup12[599033-1078174] delta 10 to telomere

Observed GCR size : 1,090 Kb

Calculated GCR size : 1.055 Kb

D11 : *rad59 tlc1* mut 7

breakpoint sequence

chromosome fusion to the Y' element of an unidentified telomere
TGTGTACTATCTTTATTTACGGAAATAAGTTGTAATA:TTAAAAAAAAAAAAAAACATTTTGATGGACAA chr V [36307]
CAAGCAACCGCATCGGCTCAACCTGATCATGCAATTA:GCAAGCAACACTGTGTACGGTACAAACAGAAA chr XVI [944773]

aCGH analysis

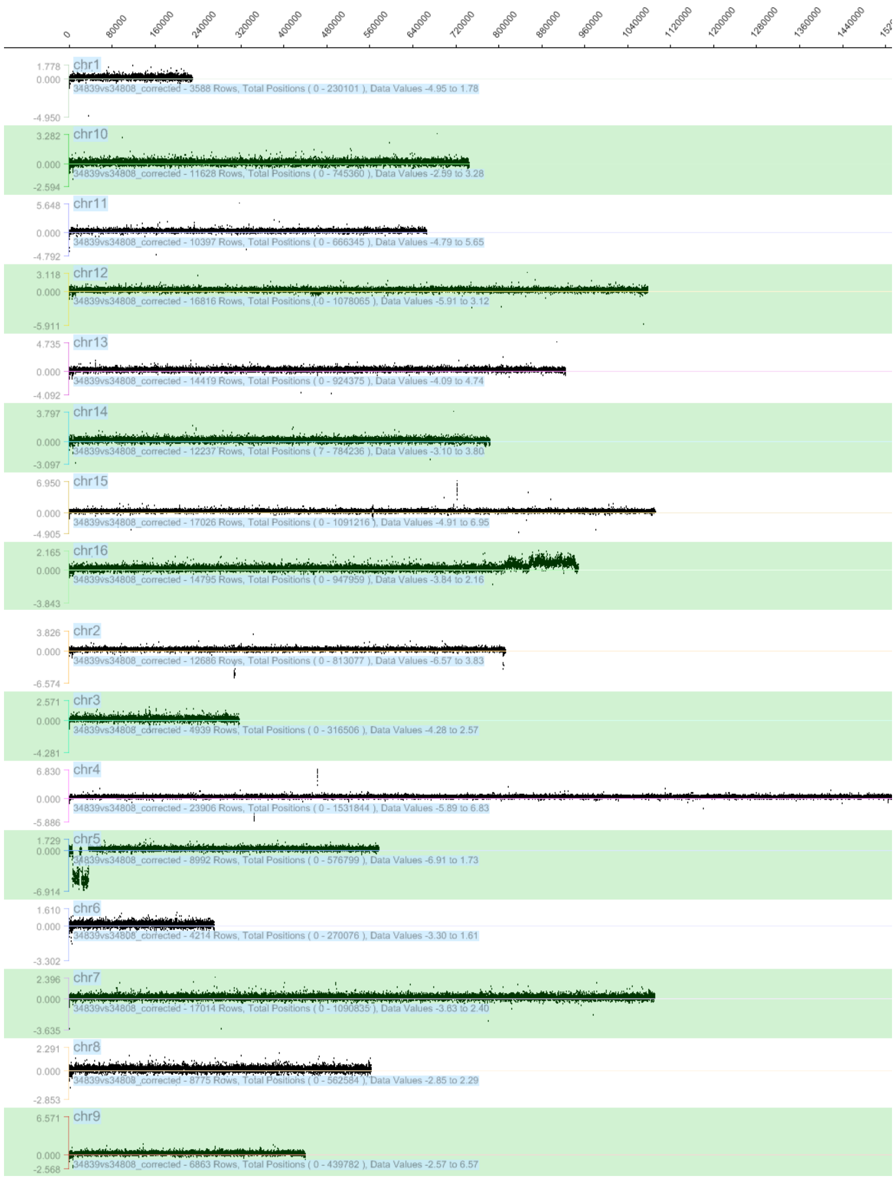

Observed karyotype modifications

del1[1-1344] L telomere
del5[1-36307] L arm
del11[1-1088] L telomere
dup16[804641-850625] delta 18 to delta 23
trip16[850625-944773] delta 23 to telomere

Observed GCR size : 800 Kb

Calculated GCR size : 778 Kb

D12 : *exo1 tlc1* mut 16

breakpoint sequence

```
dicentric translocation
TTTTAAGAGGCTTTTTGAACACTGCATTGCACCCGACAAATC:AGCCACTAACTACGAGGTCACGGACACATATACCAATAGTT chr V 42443
GCAGATATGTTTTGCTTCCACAAGAGACTCATCCAACAAATC:TTGCGTTCTGAACAAGTTTTTCTTCCTTCTTATCGCCGATG chr XIV 589827
```

aCGH analysis

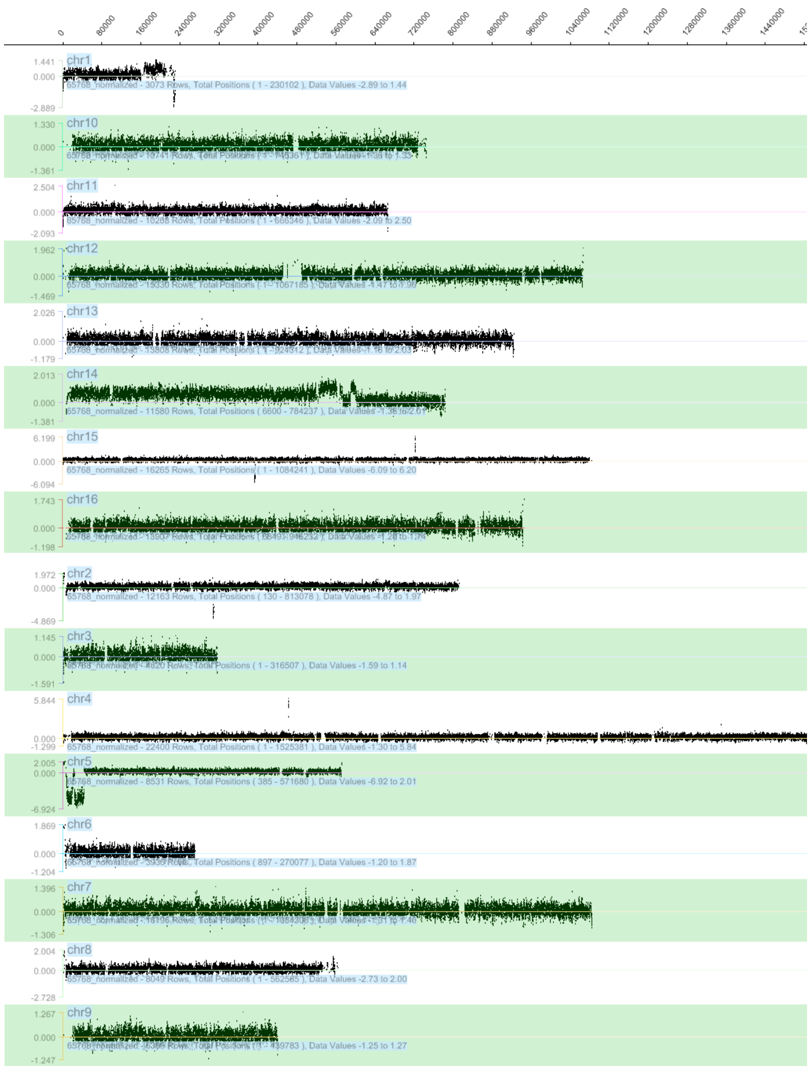

Observed karyotype modifications

del1[1-1602] L telomere
dup1[165600-222400] delta 3 to the telomere
del1[226800-230208] R telomere
del5[1-42443] L arm
dup6[1-4200] L telomere
dup7[271289-279178]
dup8[1-3000] L telomere
del11[336227-338467] *DEF1*
dup12[1065008-1078174] *YLR456* to telomere
dup14[1-519164] telomere to delta 3
tri14[519164-567993] delta 3 to delta 6
dup14[567993-574092] delta 6 to *YNL033-34W*
tri14[589827-600226] primary BP to *YNL018-19C*

Observed GCR size : 1,100 Kb

Calculated GCR size : 1,171 Kb

D13 : *tel1 tlc1* mut 13

breakpoint sequence

chromosome fusion to an unidentified telomere
TAATCTGTCGTCAATCGAAAGTTTATTTCAGAGTTCT:TCAGACTTCTTAACTCCTGTAAAAACAAAA chr V 33515
ACACCACACTACCCTAACACTACCCTAACACTACCCT:AACCCTATTCTAATCCAACCCTGATAAACC chr unidentified

aCGH analysis

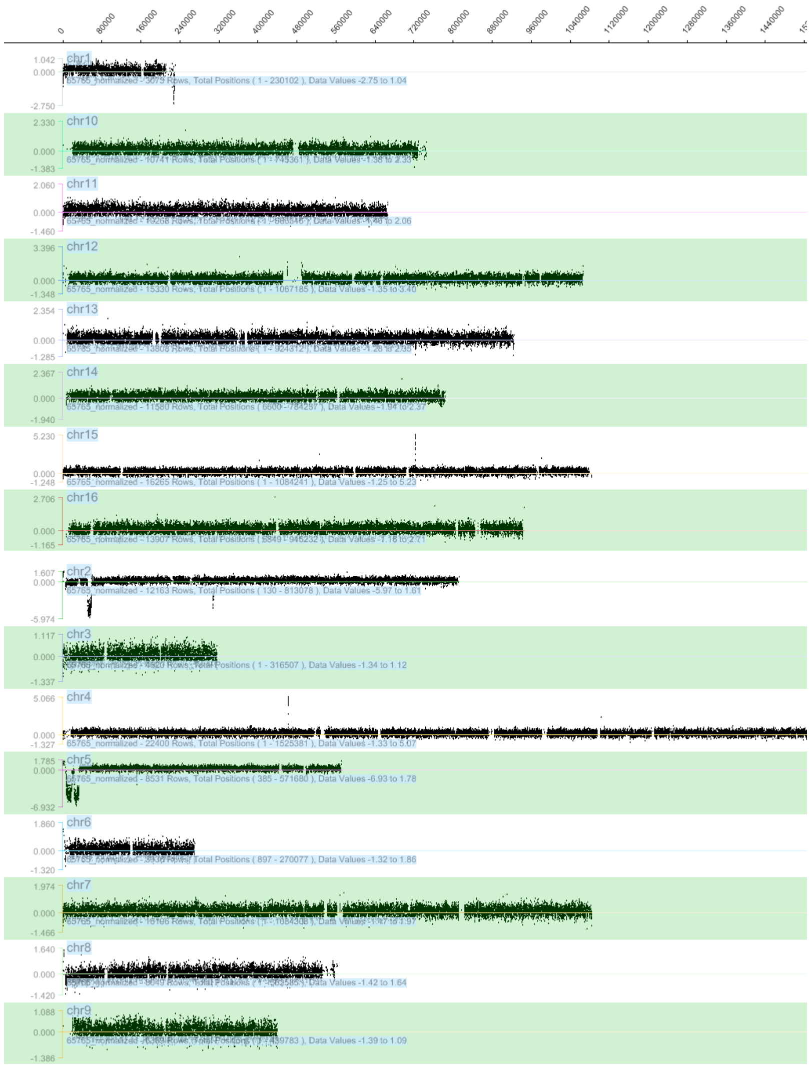

Observed karyotype modifications

del1[226900-230000]
del5[1-33515]
del5[151667-152307] centromere deletion
dup6[1-4800]
del6[4800-7616]
dup8[1-4836]

Observed GCR size : 1,500 Kb

Calculated GCR size : 1,500 Kb

D14 : *rad59 tlc1* mut 2

breakpoint sequence

first breakpoint second breakpoint

chr V [40723] CAAG**AAAAGAAAAAG:**GG**G**T**TCAA**AGGTT**C**C [40694]

chr V [40347] TGCA**AAGA**C**AAAAAG:**TA**GGTCAA**CAAAC**C**A // CACCACCAAGCGACA:TATTGGCTGTGGAAC [40698]

CACCACCAAGCGACA: poly [AC_{1-3}]n

aCGH analysis

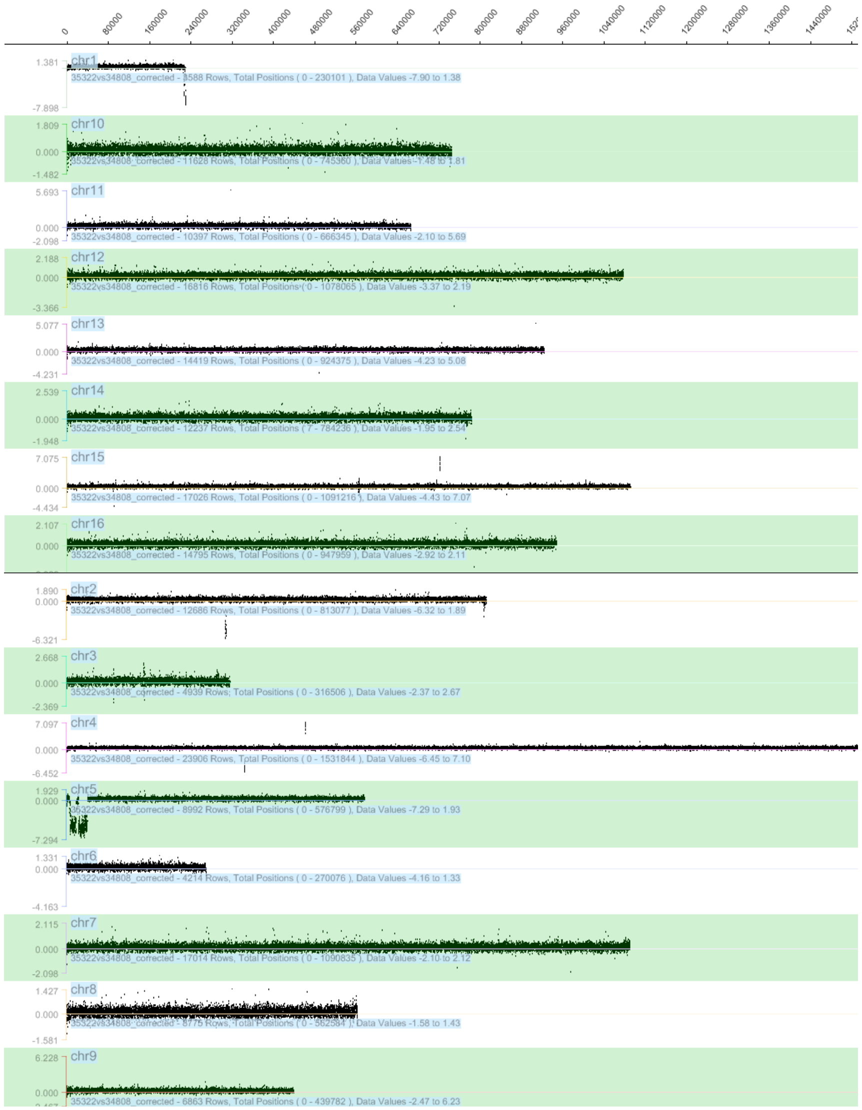

Observed karyotype modifications

del1[226812-230208] L telomere
del5[1-40291] L arm
dup5[40291-40995]

Observed GCR size : 575 Kb

Calculated GCR size : 575 Kb

D15 : *mec1 sml1 tlc1 lig4* mut 35

breakpoint sequence

dicentric translocation

TC**GCT**T**C**CAAACACTACG**G**TGT**CG**CT**AAAAATGCCAA:**CGTT**G**T**T**GCGGTGA**A**A**G**T**C**TTGAG**A**T**CA**AACGGGT chr V 40824

GT**GCT**C**C**ATGCATACGTT**G**CTG**CG**AC**AAAAATGCCAA:**GTCC**G**C**T**CAACCCG**A**T**G**C**C**CATGT**A**C**CA**CGAACAA chr XV 1050963

aCGH analysis

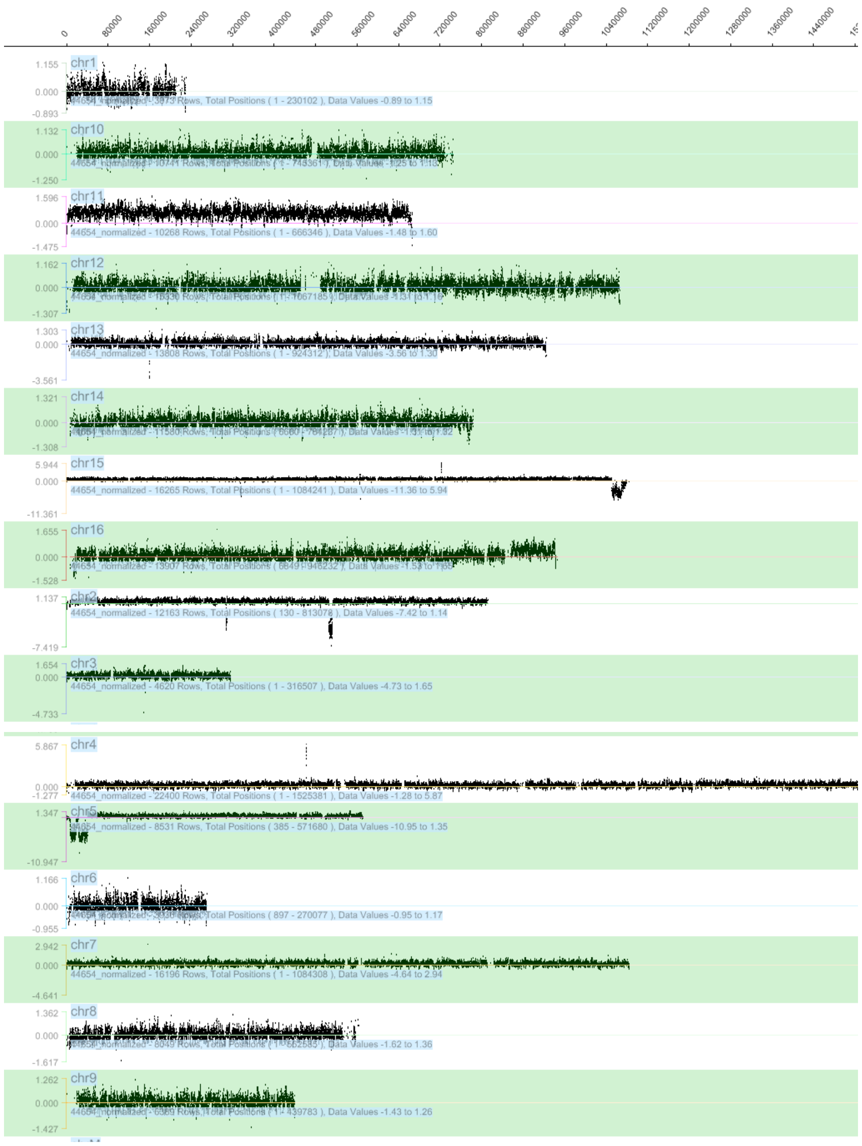

Observed karyotype modifications

del5[1-40824] L arm
dup5[40824-62081] first BP to delta1
11 disomy
del15 [1050987-1091287] from *YOR378* to telomere
include 11 non-essential genes
del16[1-19756]
dup16[844408-948062] delta 20 to R telomere

Observed GCR size : 1,500 Kb

Calculated GCR size : ND

D16 : *tel1 tlc1* mut 12

breakpoint sequence

chromosome fusion to the Y’ element of an unidentified telomere

TTAAACGAATC**T**CC**T**AAATTC**T**TCA**A**TG**T**TGTTATT**G**AGAAA:**ATC**GACATAATAAGAGTAGAT**TT**CC chr V 37414

CGCCCAACTGT**T**TT**T**TCTAGA**T**AAT**A**GA**T**AACAGAG**G**CCCCC:**ATC**TCTTAGTCTCCCTACGCC**TT**GA chr unidentified

aCGH analysis

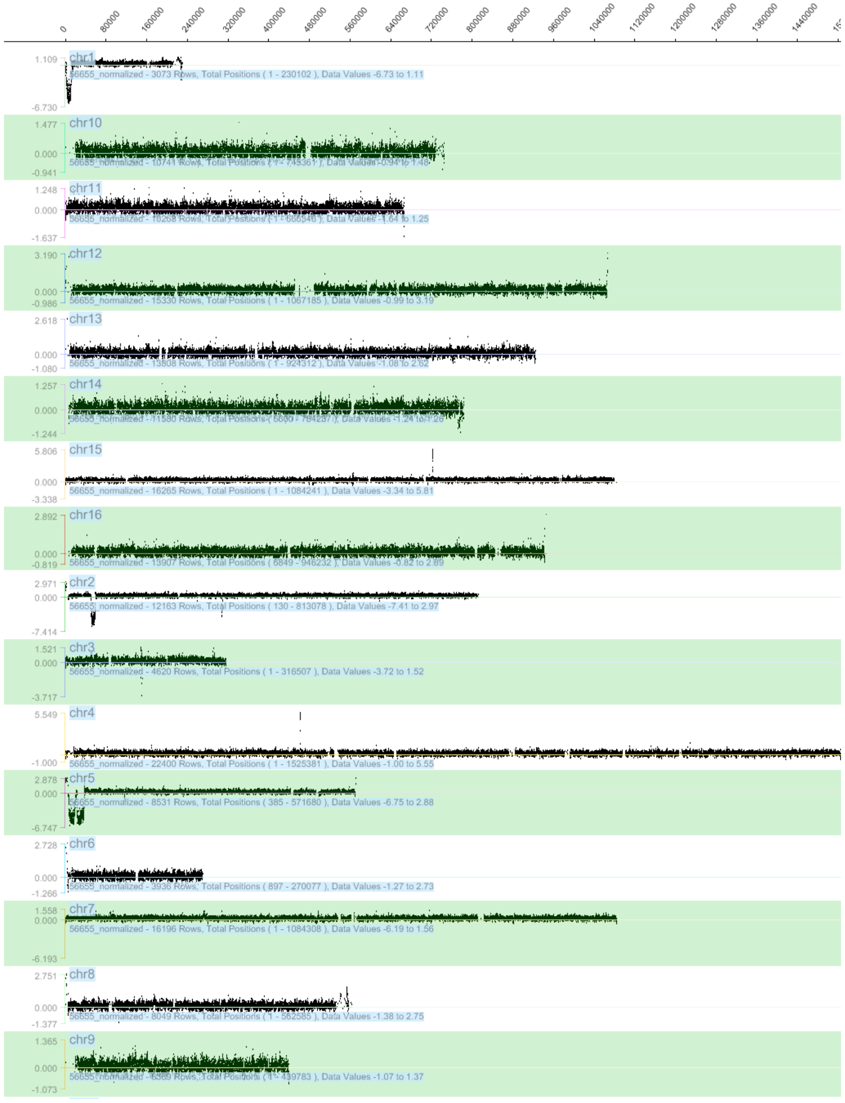

Observed karyotype modifications

del1[1-11614]
dup1[219100-226100]
del5[1-37344]
dup8[540840-556952] delta 15
dup10[462332-483619] delta 11
dup12[1065100-1078174] Y’ element
dup14[250569-256079] *MER1, SSB2, YNL208W, RIO2*
dup14[499972-503467] *RPL9B, SUN4*
del14[774346-776653]

Observed GCR size : 600 Kb

Calculated GCR size : ND

U1 : *mec1 sml1 tlc1* mut 6

breakpoint sequence

not determined

aCGH analysis

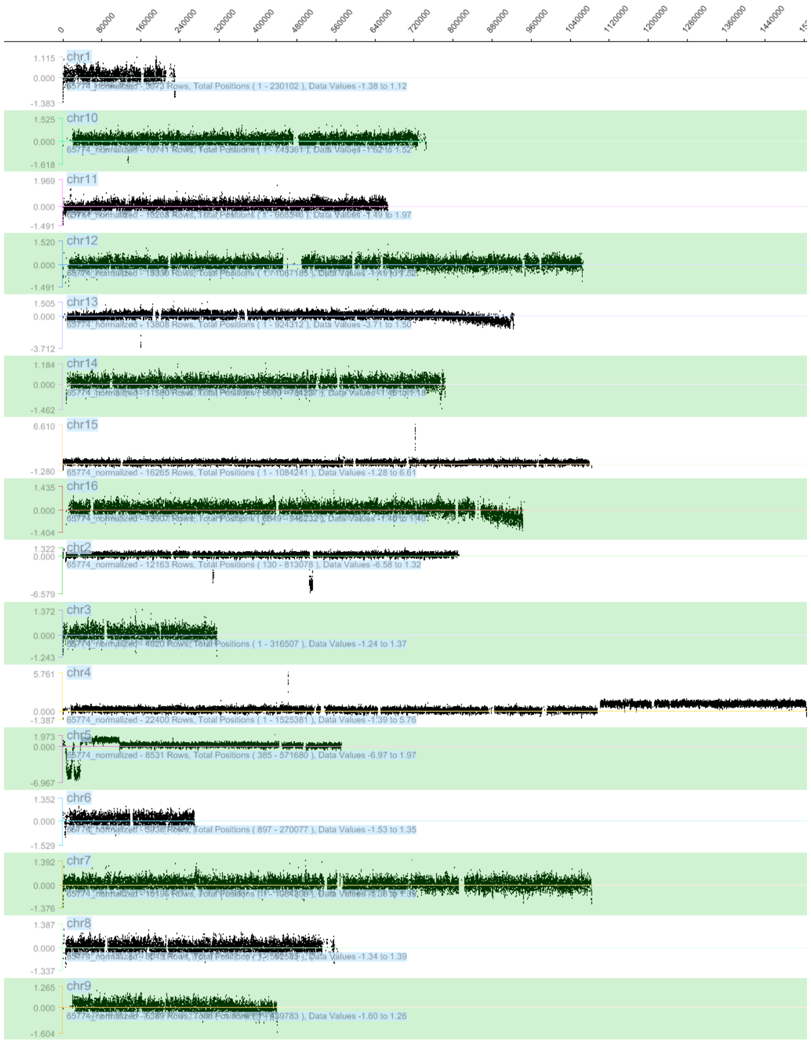

Observed karyotype modifications

del1[1-1250] L telomere
dup1[190420-192219] YAT1
dup4[1095765-1531919] from delta 23 to R telomere
del5[1-36300] L arm
dup5[36832-116167] to *URA3-52*
chromosome end decay 13[810660-924426]
chromosome end decay16[856800-948062]

Observed GCR size : 1,300 Kb

Calculated GCR size : 1,056 Kb

U2 : *mec1 sml1 lig4 tlc1* mut 28

breakpoint sequence

not determined

aCGH analysis

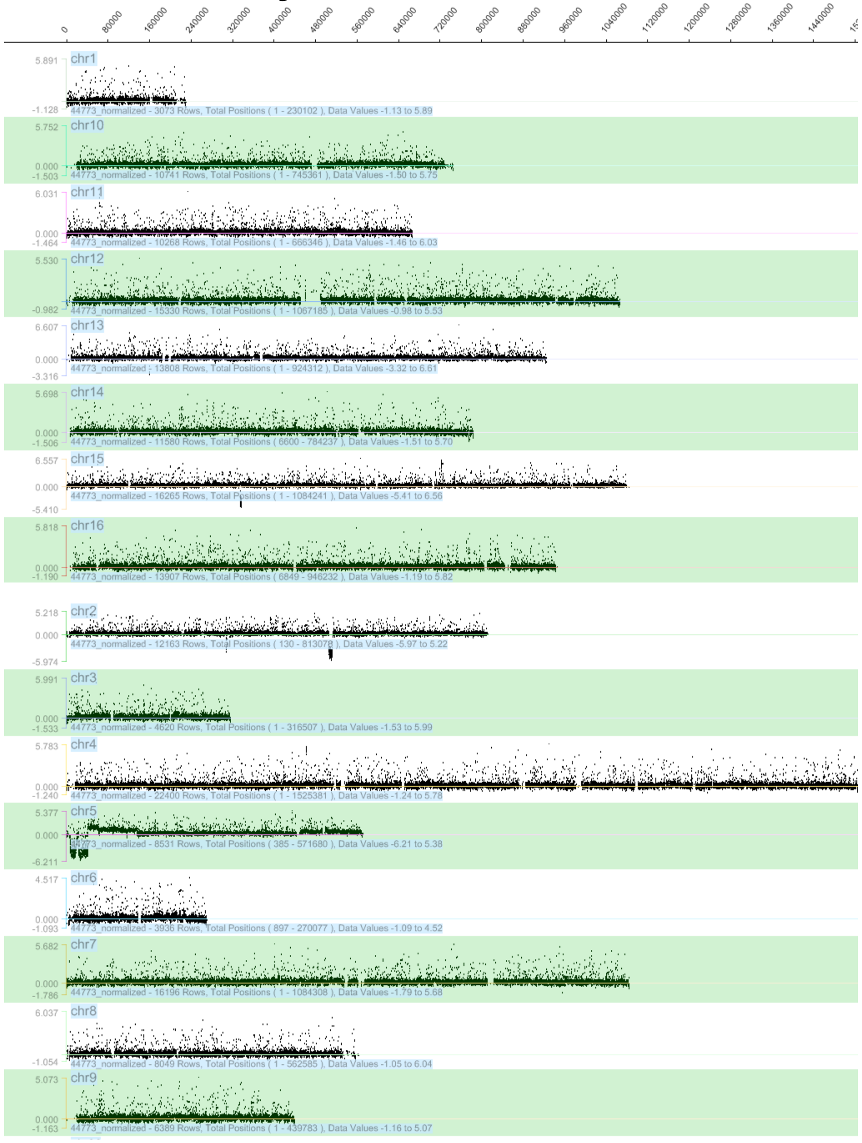

Observed karyotype modifications

del5[1-41380] L arm
trip5[41111-63728] second BP to delta 1
dup5[63728-135612] delta 1 to delta 5
dup5[443393-576869] delta 19 to R telomere

Observed GCR size : 745 Kb

Calculated GCR size : 780 Kb

U3 : *xrs2 tlc1* mut 12

breakpoint sequence

```
not determined
```

aCGH analysis

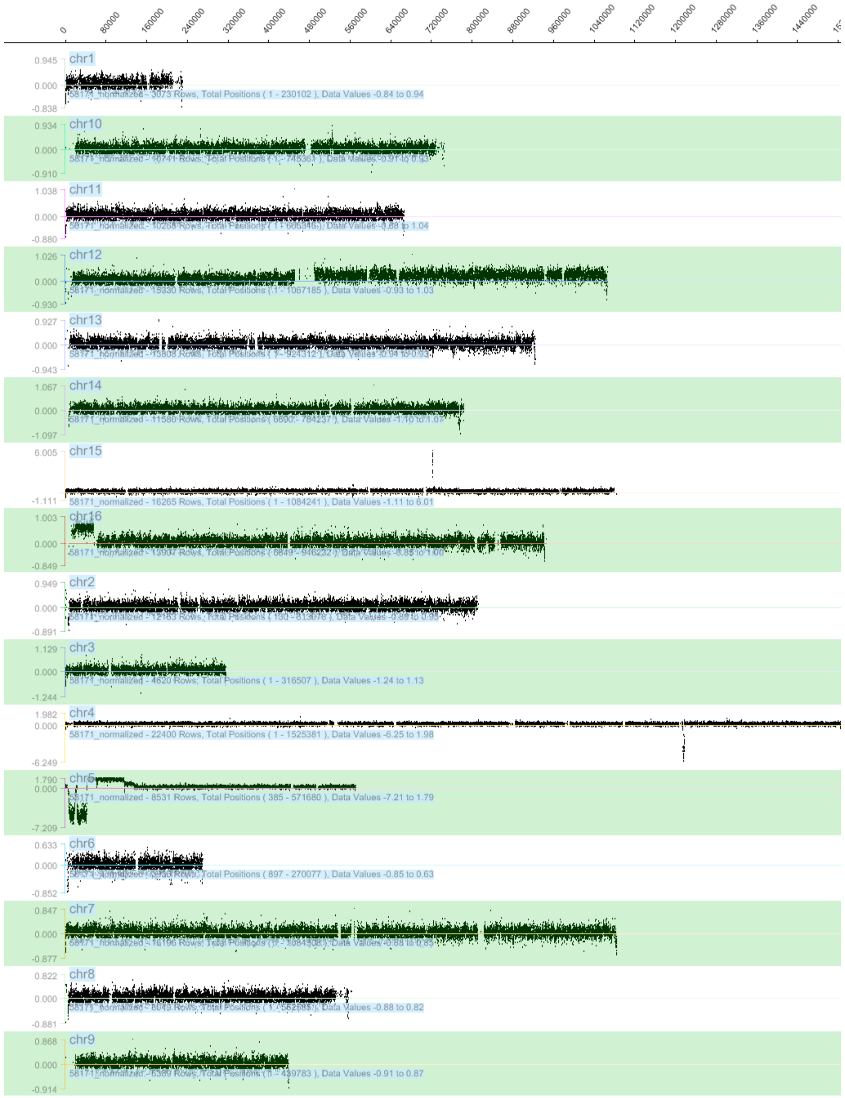

Observed karyotype modifications

del5[1-42342]
dup5[42342-42788]
quad5[42788-116167] L arm to *URA3-52*
dup5[116167-135612] *URA3-52* to delta 5
dup11[612387-614755] *SRP40*
dup12[475977-1078175] delta 7 to R telomere
dup13[886506-895400]*GAS1, PSE1, NIP1*
dup15[1070507-1091287] telomeric region
dup16[1-63006] L telomere to delta 6

Observed GCR size : 840 Kb

Calculated GCR size : 837 Kb

Karyotype modifications associated with strains mutations

del2[505520-512757] *MEC1* deletion
del2[50995-59315] *TEL1* deletion
del2[307493-308965] *TLC1* deletion

dup4[467120-4625509] *TRP1 insertion*
del4[344078-344974] *RAD59* gene
del4[598411-599765] *RAD55* deletion
del4[1214980-1217550] *XRS2* deletion

del5[115827-117062] *URA3* gene
del5[349908-351572] *RAD51* deletion

del13[159193-154769] *SML1* deletion

del15[334454-339334] *LIG4* deletion
dup15[721699-722787] *HIS3* insertion
del15[392000-395000] *EXO1* deletion
